# Stealthy Sensor Attacks on IoT-Enabled Model Predictive Control of Perishable-Goods Production: Residual-Based Detection, Mitigation, and Secure Sensing Design

**DOI:** 10.3390/s26216920

**Published:** 2026-10-31

**Authors:** Lotfi Tadj

**Affiliations:** College of Engineering and Advanced Computing, Alfaisal University, Riyadh 11533, Saudi Arabia; ltadj@alfaisal.edu

**Keywords:** IoT security, false data injection, replay attack, Kalman filter, CUSUM, model predictive control, cyber-physical systems, industrial IoT, perishable inventory, resilient control

## Abstract

IoT-enabled model predictive control (MPC) lowers cost and spoilage in perishable-goods production but depends on sensor data that an attacker can falsify, which network intrusion detection cannot verify. We study sensor attacks on a published IoT-MPC plant model and a defense that needs no additional model: a CUSUM test on the innovations of the Kalman filter already in the loop, isolation of flagged sensors, and re-estimation from the attack onset. With accurate sensors and an exact model, a falsified energy meter raises the tracking cost by 71% and replay raises spoilage by 27%; the defense detects them in 89% and 100% of runs at a nominal 1% false-alarm rate and limits the cost increases to 9% and 3%. In closed loop, static detectors miss moderate and slow attacks that predictive detectors find. Detection weakens with sensor noise and model error; a fully informed attacker raises the cost by 93% undetected, but model errors expose it, and a redundant meter on an independent path limits it to about 15%. On the HAI testbed, an identified-model residual detector finds 32 of 34 attacks at 0.24 false alarms per day, against 27 for a windowed autoencoder and 21 for a static range check.

## 1. Introduction

Producers of perishable goods, such as fresh food, dairy and pharmaceuticals, operate with short shelf lives, tight margins and energy-intensive storage. Internet of Things (IoT) technologies now let them monitor inventory, storage conditions, energy use and equipment health in real time and feed these measurements into the control of production. In earlier work we developed a model predictive control (MPC) framework for perishable-goods production that couples inventory, machine condition, energy use, demand forecasts, predictive technology interventions and spoilage, and we showed that fusing IoT measurements with model predictions through a Kalman filter lowers both cost and spoilage compared with model-only control or with raw sensor data [1,2].

The same data pipeline that makes this possible also creates an attack surface. Readings travel from low-power wireless sensors (for example over LoRaWAN, a long-range wide-area network protocol whose security weaknesses are documented [3]) through edge and cloud layers to the controller, whose commands return to the plant over industrial protocols. Manufacturing has been the most attacked industry in IBM’s threat intelligence reports for four consecutive years up to the 2025 edition [4], and food producers have been hit directly: a ransomware attack in 2021 forced the meat processor JBS to halt plants [5], and Dole reported a ransomware incident in 2023 [6]. Those incidents disrupted information systems. A less visible threat is the falsification of sensor data. A spoofed energy meter or a replayed temperature log does not stop the plant; it steers the controller into costly decisions while every reading looks plausible. The same effect can arise from an insider who falsifies cold-chain records to pass an audit.

Research on IoT intrusion detection has focused mainly on classifying network traffic with machine-learning and deep-learning models trained on benchmark datasets [7,8,9]. These detectors look at packets, not at the physical process, and they cannot tell that a well-formed packet carries a false value. Control-theoretic research on cyber-physical systems offers a complementary approach, detection based on a model of the process [10,11,12,13,14], but it has been developed mostly for power grids, water systems and chemical processes. We are not aware of a study that examines how sensor attacks affect IoT-enabled control of production and inventory with perishability, how well the estimator such systems already contain can detect and mitigate them, or how the sensing design changes what an attacker can achieve.

This paper is an application and empirical study that addresses these questions. It uses the published IoT-MPC model of [1], re-implemented from its original MATLAB R2025a (MathWorks, Natick, MA, USA) code, and it validates the detection approach on a real industrial testbed. Its contributions are as follows:

1. A threat model with four levels of attacker knowledge and a set of sensor attacks (bias, ramp, intermittent bias, replay, denial of service and detector-aware attacks with full or imperfect knowledge) for IoT-enabled MPC of perishable-goods production, evaluated by tracking cost, spoilage and energy with confidence intervals. The evaluation ranks the attack surface: the energy meter is the most valuable single target for raising cost under the three cost weightings tested, while fixed-shape attacks on the inventory sensor mainly raise spoilage.
2. A lightweight defense that reuses the Kalman filter already in the loop: a per-sensor cumulative-sum (CUSUM) test on the innovations, calibrated to a fixed false-alarm rate, combined with isolation of flagged sensors and a rewind-and-refilter repair of the state estimate. We show how quickly its power falls when the plant differs from its model, and when a bounded-memory $\chi^{2}$ statistic should replace CUSUM.
3. Evidence, in simulation and on the HAI (hardware-in-the-loop-based augmented industrial control system) testbed [15], that static anomaly detectors miss moderate and slow attacks in closed loop because feedback keeps falsified readings within their normal range, whereas detectors that predict the dynamics, whether from a first-principles model, an identified linear model or a learned neural network, detect them. On HAI, where we test predictive-residual detection with an identified model and a bounded-memory statistic rather than the full defense, it finds 32 of 34 real attacks at 0.24 false alarms per day, against 27 for a windowed autoencoder, 21 for a static range check and 2 for an Isolation Forest under the same protocol.
4. An analysis of the damage that remains possible, for fixed-shape attacks kept small enough to go unnoticed and for detector-aware attackers that watch one or all detector channels, together with an explanation, from the filter’s transfer function, of why some sensors are much harder to protect than others. With noisy sensors, faster sampling and more precise sensors do not reduce this damage, whereas a redundant sensor on the most valuable measurement does, provided it is not reachable through the same compromised network path; if it is, redundancy increases the damage. Robustness checks cover the detector threshold, measurement noise, plant-model mismatch, packet loss, intermittent attacks and imperfect attacker knowledge.

The detection and estimation tools used here, Kalman-filter innovations, CUSUM and sensor isolation, are classical, and we use them deliberately: the defense adds no model and almost no computation to an architecture that already contains the filter. The novelty lies in what the study shows with them. First, it analyzes the security of an IoT-enabled production and inventory controller, in which attacks act through the control loop on cost and spoilage rather than on a physical safety limit, as in the power, water and chemical processes studied so far. Second, it explains, with open-loop and closed-loop experiments and with real testbed data, why static detectors fail in such a loop while predictive ones, model-based or learned, do not. Third, it derives from the filter’s transfer function which sensors are hard to protect, before any attack is simulated. Fourth, it compares sensing designs against fixed-shape, fully informed and imperfectly informed attackers, and shows when redundancy helps and when it increases the damage.

The rest of the paper is organized as follows. Section 2 reviews related work. Section 3 recalls the plant and controller and defines the threat model. Section 4 presents the detectors and the mitigation scheme. Section 5 reports the simulation results and the validation on HAI, Section 6 discusses their implications and limitations, and Section 7 concludes. Appendix A lists all model and controller parameters, Appendix C the settings of all detectors, and Appendix B the main symbols.

## 2. Related Work

### 2.1. Intrusion Detection for the IoT

Intrusion detection for the IoT has become a large field, reviewed in several recent surveys [7,8,9]. Most proposed detectors are machine-learning or deep-learning classifiers of network traffic, evaluated on benchmark datasets such as NSL-KDD, UNSW-NB15, CIC-IDS2017 or TON_IoT. The papers published so far in this Special Issue are representative: they address class imbalance with generative augmentation [16], detect volumetric denial-of-service attacks with attention-based recurrent networks [17], and combine metaheuristic feature selection with deep ensembles [18]. Such detectors are effective against attacks that change traffic patterns, but they observe the network, not the process, and cannot recognize a correctly formatted reading that carries a false value. Their evaluations also rest on labeled attack traffic, which is rarely available for a specific plant, and they often report accuracies close to 100% on datasets in which attack and normal traffic are easy to separate. Network-level and process-level detection are therefore complementary. Data-driven detectors have also been applied to process data from industrial testbeds, for example convolutional networks on the Secure Water Treatment (SWaT) dataset [19], recurrent networks that predict the next sensor values [20], and autoencoders over windows of multivariate data [21]. These process-level studies usually score detectors point by point, a practice that can overstate their performance on time series [22], and they evaluate detection without the controller that reacts to falsified readings. Whether such a detector works in closed loop depends, as we show, on whether it predicts the dynamics or only models the distribution of individual readings.

### 2.2. Attack Detection in Cyber-Physical Systems

Detecting abnormal behavior from the innovations of a state estimator is a classical idea in fault detection [23,24]. Its security counterpart must deal with an adversary. Liu et al. showed that false data injection can bypass the bad-data detection of power-system state estimators [25]; Mo and Sinopoli showed that replay attacks can defeat residual detectors and proposed watermarking the control input to expose them [10], and later bounded the performance degradation that stealthy integrity attacks can cause [26]. Kwon et al. derived conditions under which deception attacks remain stealthy [27], Pasqualetti et al. characterized which attacks are fundamentally undetectable in linear systems [11], and Fawzi et al. showed how to estimate the state when a subset of sensors is compromised [28]. Teixeira et al. organized attacks by the adversary’s knowledge, disclosure and disruption resources [12], and Cárdenas et al. studied attacks, detection and response on a chemical process model [29]. Urbina et al. argued that detectors should be compared by the damage an undetected attacker can still cause at a given false-alarm rate, and found stateful detectors such as CUSUM preferable to stateless ones on industrial testbeds [13]; Murguia and Ruths analyzed CUSUM and $\chi^{2}$ detectors for compromised sensors [30]; Umsonst et al. compared both by the worst-case impact of stealthy attacks [31] and studied how to tune detectors against them [32]; and Murguia et al. proposed security metrics and a synthesis method for secure controllers and monitors [33]. Giraldo et al. survey this literature [14]. Our evaluation follows this impact-based view: the detector-aware attacker of Section 3.3 is the greedy stealthy attacker of [13,31], and our finding that CUSUM limits it better than a $\chi^{2}$ statistic agrees with theirs. Most of these analyses assume an attacker who knows the model, the detector and its state exactly, and they evaluate detectors at a single operating point; how much an imperfectly informed attacker can achieve, and how the trade-off between false alarms and damage looks across thresholds, receive less attention. What these studies do not cover at all is production and inventory control with perishable goods, where the consequences of an attack are economic and accumulate through spoilage, the behavior of data-driven detectors in such a loop, and how the sensing design changes what an attacker can achieve.

### 2.3. IoT-Enabled Control of Perishable Production

Inventory models with deterioration have a long history in operations research [34], and MPC is increasingly used for energy-intensive operations such as cooling [35]. Our earlier work combined these strands in an IoT-enabled MPC framework for perishable-goods production and compared model-only control, direct use of IoT readings, and Kalman-filtered IoT integration [1,2]. That work, like the related literature on perishable production, assumed that sensor data are noisy but honest. This paper removes that assumption.

## 3. System Model and Threat Model

### 3.1. IoT-Enabled MPC of Perishable-Goods Production

We build on the IoT-enabled MPC framework of [1,2], summarized here only as far as needed for the security analysis; all parameters are listed in Appendix A. The plant is described by seven coupled states, $x={\left[I,M,E,F,P,A,S\right]}^{⊤}$: inventory, machine performance, energy consumption, demand forecast, production rate, the level of predictive technology interventions (PTI), and spoilage rate, all in the normalized units of [1]. Five inputs $u\in R^{5}$ act on maintenance, energy management, forecast correction, production adjustment and PTI. Discretizing the linear model with forward Euler and a step of h=0.1 (in hours) gives

(1) $$x_{k+1}=A_{d}x_{k}+B_{d}u_{k}+w_{k},\;y_{k}=Cx_{k}+v_{k},$$

with $A_{d}=I+hA$ (I denotes the identity matrix), $B_{d}=hB$, process noise $w_{k}∼N\left(0,Q_{w}\right)$ and measurement noise $v_{k}∼N\left(0,R_{v}\right)$. Three IoT sensors are available: a radio-frequency identification (RFID) inventory count, an energy meter, and a spoilage index derived from temperature and humidity readings, so that *C* selects *I*, *E* and *S*.

The controller tracks time-varying targets $x_{k}^{\mathrm{ref}}$ (inventory and production ramping up, a sinusoidal energy profile, decaying forecast, PTI active for the first 5 h, slowly rising spoilage allowance). Feedforward inputs $u_{k}^{\mathrm{ref}}$ are obtained by inverting the dynamics and clipping at zero, and the MPC acts on the deviations $\Delta x_{k}={\hat{x}}_{k}-x_{k}^{\mathrm{ref}}$ over a horizon of m=5 steps with the weights of [1]. As in [1], the quadratic program is solved without constraints and its solution, $\Delta u_{k}=K_{\mathrm{MPC}}\;\Delta x_{k}$, is saturated componentwise, $u_{k}={\mathrm{clip}}_{\left[0,10\right]}\left(u_{k}^{\mathrm{ref}}+\Delta u_{k}\right)$; the controller is therefore a saturated linear state feedback. The simulated horizon is 10 h (101 steps).

The three architectures of [1] differ only in the state estimate ${\hat{x}}_{k}$ passed to the controller. Scenario A (no IoT) propagates the model open loop. Scenario B (raw IoT) overwrites the measured entries of the predicted state with the latest readings and propagates the model between readings. Scenario C (IoT with Kalman filtering) fuses readings and predictions:

(2) $$\begin{array}{cccc} {\hat{x}}_{k|k-1} & =A_{d}{\hat{x}}_{k-1|k-1}+B_{d}u_{k-1}, & \Sigma_{k|k-1} & =A_{d}\Sigma_{k-1|k-1}A_{d}^{⊤}+Q_{w}, \end{array}$$

(3) $$\begin{array}{cccc} r_{k} & =y_{k}-C{\hat{x}}_{k|k-1}, & V_{k} & =C\Sigma_{k|k-1}C^{⊤}+R_{v}, \end{array}$$

(4) $$\begin{array}{cccc} {\hat{x}}_{k|k} & ={\hat{x}}_{k|k-1}+K_{k}r_{k}, & K_{k} & =\Sigma_{k|k-1}C^{⊤}V_{k}^{-1},\;\Sigma_{k|k}=\left(I-K_{k}C\right)\Sigma_{k|k-1}. \end{array}$$

When no reading arrives, the update step is skipped. The innovation $r_{k}$ and its covariance $V_{k}$ are the basis of the detector in Section 4.

**Sensing regimes.** We use the two regimes of [1]. In Experiment 1 (good sensors) $Q_{w}=5\times 10^{-3}I$, $R_{v}=\mathrm{diag}\left(0.05,0.05,0.02\right)$ and all sensors report every step. In Experiment 2 (noisy, sparse sensors) $Q_{w}=10^{-2}I$, $R_{v}=\mathrm{diag}\left(0.8,0.8,0.4\right)$ and readings arrive every fourth step (every 0.4 h).

**Reproduction of the baseline.** All simulations use a Python 3.13 (Python Software Foundation, Wilmington, DE, USA) re-implementation of the MATLAB R2025a, MathWorks, Natick, MA, USA code behind [1]. Table 1 compares its Monte Carlo costs with the published ones. The differences are within about two standard errors, which is expected because the two implementations draw different random numbers, and the ranking of the scenarios is preserved in both experiments. The code is available from the author on request (Data Availability Statement).

### 3.2. Adversary Model

**Figure 1:**
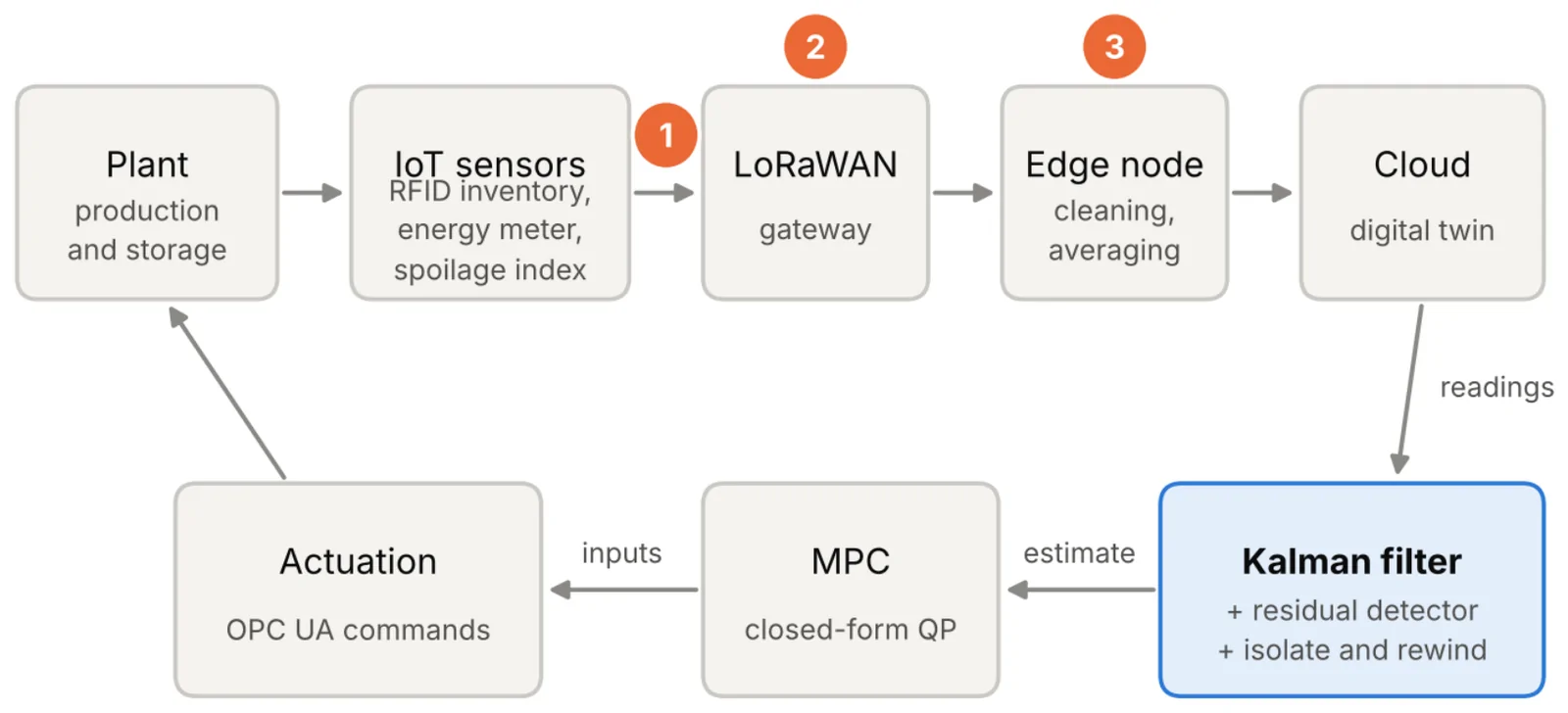
IoT-enabled MPC architecture of [1] and the points where the adversary can alter, replay or suppress sensor readings: (1) a rogue or cloned sensor node or radio jamming, (2) a compromised gateway or stolen network keys, (3) an insider at the edge node. The controller, the actuator commands and the plant are not attacked. The Kalman filter with the residual detector and the isolate-and-rewind mitigation (blue) is the defense studied in this paper.

The IoT pipeline of [1] carries readings from wireless sensors (LoRaWAN) through an edge node and a cloud digital twin to the MPC, and returns control commands to the plant over an industrial protocol (OPC Unified Architecture, OPC UA). We consider an adversary who can read, alter, replay or suppress sensor packets somewhere between the sensors and the estimator: at a sensor (a rogue or cloned node, or radio jamming), at the gateway (a compromised gateway or stolen network keys), or at the edge node (an insider). The adversary cannot alter the controller, the actuator commands or the plant itself. A node-level attacker reaches only the sensors it controls, whereas a gateway or edge attacker can reach every sensor behind that gateway or node; this distinction matters for the redundancy results of Section 5.4. Figure 1 shows the architecture and the points of attack. The attacker’s goal is to raise cost, spoilage or energy use while avoiding detection.

We consider four levels of attacker knowledge, summarized in Table 2. A *fixed-shape* attacker knows which sensor feeds which state and can record clean readings before attacking, but does not observe the detector; for this attacker we vary the attack magnitude to find the most damaging attack that still goes unnoticed. A *detector-aware* attacker additionally knows the plant model, the Kalman filter state, the detector statistics and thresholds, and the clean readings of the sensors it controls (including their measurement noise), as in the worst-case analyses of [13,31]; it may watch only its own sensor’s statistic or the statistics of all sensors. These assumptions give an upper bound on what a greedy attacker can achieve, but they are strong: the filter state and the detector state live inside the edge node or the controller. An attacker with *imperfect knowledge* therefore observes only the readings that pass through the compromised gateway and the control commands, and runs its own replica of the filter with an approximate model and an estimate of the threshold. The target-aligned replay below also assumes that the attacker knows the targets, for example from recorded setpoints.

### 3.3. Attack Models

From the attack time $k_{a}$ onward (at $t_{a}=3$ h unless stated otherwise), the estimator receives ${\tilde{y}}_{k}$ instead of $y_{k}$:

- **Bias (false data injection):** ${\tilde{y}}_{k}=y_{k}+e_{i}b$, a constant offset *b* on sensor *i* ($e_{i}$ is the *i*-th unit vector).
- **Ramp:** ${\tilde{y}}_{k}=y_{k}+e_{i}\;s\;\left(t_{k}-t_{a}\right)$, a slowly growing offset that mimics sensor drift and is designed to evade detection.
- **Replay:** The attacker records the clean readings of all sensors during the 4 h before $t_{a}=4$ h, which is one full period of the energy target, and replays them in a loop.
- **Target-aligned replay:** As replay, but each replayed reading is shifted by the change in its target since it was recorded, ${\tilde{y}}_{k}=y_{k^{\prime }}+C\left(x_{k}^{\mathrm{ref}}-x_{k^{\prime }}^{\mathrm{ref}}\right)$, where $k^{\prime }$ is the recorded step, so that the replayed readings follow the current targets. Both replays are also started at $t_{a}=6$ h, so that the recording (2 to 6 h) excludes the start-up period.
- **Intermittent bias:** The bias is switched on for a fraction *d* (the duty cycle) of each period *T* and off for the rest, which mimics an attacker who tries to keep the detector statistic from accumulating.
- **Denial of service (DoS):** Each reading is dropped independently with probability 0.8. In the robustness checks of Section 5.6, random packet loss is also combined with the other attacks.
- **Short, large bias:** A bias of −2 or −3 applied for only 0.5 h, designed to trigger an alarm and thereby the isolation of the sensor.
- **Detector-aware attack, own channel:** At each reading the attacker sets the normalized innovation of the targeted sensor to the largest value, in the damaging direction, that keeps that sensor’s detector statistic at least 0.5 below its threshold. For CUSUM this means $|z_{i,k}|=\max\left(0,\tau -0.5-g_{i,k-1}+\nu \right)$ with the notation of Section 4, where k−1 denotes the sensor’s previous reading (four steps earlier in Experiment 2); a zero push means that the attacker sends the value the filter predicts. For the windowed statistic the bound applies to the window mean, and with an additional per-reading test $|z_{i,k}|$ is also kept 0.5 below $\sqrt{\tau_{1}}$.
- **Detector-aware attack, full bank:** As above, but the attacker also watches the statistics of the sensors it does not control (the uncompromised sensors). Before each reading it predicts, without noise and over the next 20 readings, how its injection will shift their innovations through the state estimate, assuming the attack then continues at the drift rate, and it chooses the largest push for which none of their statistics is predicted to come within 0.5 of its threshold (and, optionally, none of their normalized innovations to exceed a bound $\bar{z}$). If no push is safe, it chooses the one that is predicted to come closest. This attacker is still greedy: it optimizes one reading at a time.
- **Detector-aware attack, imperfect knowledge:** As the own-channel attack, but the attacker computes the predicted reading and its own CUSUM statistic from a replica filter that uses a model whose nonzero parameters carry independent relative errors with standard deviation $\epsilon_{a}$, starts accumulating its statistic only when the attack begins, and assumes the threshold $\tau_{a}=f\tau$. A conservative attacker uses f<1.

Biases and ramps are reported with the sign that harms the plant; negative values mean that the sensor under-reads. An energy meter that under-reads, for instance, leads the controller to raise energy input. The opposite sign can lower the tracking cost. In the controller of [1] the targets lie below the plant’s natural trajectory, so the inputs sit at their lower bound of zero in 90 to 97% of attack-free steps and the energy level runs on average 0.8 above its target; an over-reading energy meter therefore moves energy towards its target. We report damaging directions only.

### 3.4. Performance Measures

Performance is measured by the tracking cost of [1],

(5) $$J=h\sum_{k}\frac{1}{2}\left(\Delta x_{k}^{⊤}Q\;\Delta x_{k}+\Delta u_{k}^{⊤}R\;\Delta u_{k}\right),$$

with the weights *Q* and *R* of Appendix A, evaluated on the true state and the applied (clipped) inputs over the whole 10-h horizon, without the terminal weight of the controller, together with the mean spoilage rate and mean energy level. *J* is a quadratic penalty on deviations from the operating targets, not a monetary cost. Because it also accumulates over the 3 h before the attack, percentage increases in *J* understate the effect of an attack on the period after its onset; for example, the energy bias discussed in Section 5.1 raises *J* by 71% over the horizon and by 99% over the period after onset.

Detection performance is measured by the detection probability $P_{D}$, the share of runs whose first alarm, on any sensor, occurs at or after the attack starts (a run whose first alarm precedes the attack counts as not detected; there were no such runs in Table 3; Appendix C separates alarms on the attacked sensor from alarms on other sensors), the detection delay, and the false-alarm rate (FAR), the share of attack-free runs with any alarm. To compare detectors with the measures used in intrusion detection, we also treat each 10-h run as one case, attacked or attack-free, and report recall ($P_{D}$), precision $P_{D}/\left(P_{D}+\mathrm{FAR}\right)$ for equal numbers of attacked and attack-free runs, and their harmonic mean, the F1 score. Every configuration is evaluated over the same 100 Monte Carlo seeds, so that comparisons between architectures and attacks are paired. We report 95% Wilson intervals for detection probabilities and 95% paired bootstrap intervals (2000 resamples of the seeds) for relative cost increases.

Two measures summarize how much damage remains possible against a fixed-shape attacker. The *stealthy damage* is the largest relative cost increase within an attack family that is detected in at most a fraction ε of runs,

(6) $$D_{\varepsilon }=\max_{a\in A:\;P_{D}\left(a\right)\leq \varepsilon }\;\frac{J\left(a\right)-J_{0}}{J_{0}},\;\varepsilon =0.05,$$

where A is a grid of magnitudes within one attack family (biases from 0.125 to 2 in steps of 0.125, or ramp slopes from 0.05 to 0.6 per hour in steps of 0.05, on one sensor), J(a) is the undefended cost and $J_{0}$ is the attack-free cost. It is an empirical lower bound on what a stealthy attacker can achieve, since the attacker is restricted to fixed shapes; the detector-aware attacks of Section 3.3 give a worst case among greedy attackers. The *worst-case defended damage* is $\max_{a\in A}\left[J_{\mathrm{def}}\left(a\right)-J_{0}\right]/J_{0}$, where $J_{\mathrm{def}}$ is the cost with detection and mitigation active; it captures attacks that are detected, but too late to prevent harm. Both measures carry bootstrap intervals that account for the maximum over the grid.

## 4. Detection and Mitigation

### 4.1. Residual Generation

Following the classical innovations approach to fault detection [23,24], the detector reuses the Kalman filter of Scenario C, so it needs no additional model and no attack data. At each received reading, the innovation of every sensor is normalized by its predicted standard deviation,

(7) $$z_{i,k}=\frac{r_{i,k}}{\sqrt{{\left[V_{k}\right]}_{ii}}}.$$

Without attack, the $z_{i,k}$ are white with zero mean and unit variance if the model is correct, which holds in Section 5.1, Section 5.2, Section 5.3 and Section 5.4 because the simulated plant is the filter’s model. Over 1000 attack-free runs we measured standard deviations of 0.99 to 1.01 and lag-one autocorrelations of at most 0.02 in absolute value in both experiments. An attack that shifts a reading shows up as a shift in the mean of the corresponding $z_{i,k}$, at least until the filter absorbs it into the state estimate. Section 5.5 examines what happens when the plant differs from the model.

### 4.2. CUSUM Detector

Each sensor has a two-sided cumulative-sum (CUSUM) test [36]:

(8) $$g_{i,k}^{+}=\max\;\left(0,\;g_{i,k-1}^{+}+z_{i,k}-\nu \right),\;g_{i,k}^{-}=\max\;\left(0,\;g_{i,k-1}^{-}-z_{i,k}-\nu \right),$$

and an alarm is raised on sensor *i* when $\max(g_{i,k}^{+},g_{i,k}^{-})>\tau$. The drift ν=0.5 tunes the test to mean shifts of about one innovation standard deviation. The threshold τ is set so that 1% of 10-h attack-free runs raise any alarm, estimated from 1000 attack-free Monte Carlo runs with seeds separate from those used for evaluation; this gives τ=8.5 in Experiment 1 and τ=6.5 in Experiment 2. A quantile estimated from 1000 runs rests on about ten exceedances, so the threshold is uncertain: a bootstrap over the calibration runs gives 95% intervals of 8.0 to 8.8 and 6.2 to 6.9, and on 7000 attack-free runs (the calibration runs and 6000 fresh ones) the rounded thresholds give false-alarm rates of 1.5% (95% Wilson interval 1.2 to 1.8%) and 1.6% (1.4 to 2.0%). The 1% rate quoted in the rest of the paper is therefore the calibration target, and the effective rate of the nominal thresholds is about 1.5% per run. All detection probabilities are conditional on thresholds with this uncertainty. The rate is per 10-h run, about 2.4% per day at the target rate and 3.6% at the effective rate; with permanent isolation, months of continuous operation would eventually remove sensors after false alarms, so a deployment needs a lower per-run rate or a supervised re-admission procedure. Because each sensor has its own statistic, an alarm usually points to the sensor under attack. Against the detector-aware attackers, Section 5.2 also tests CUSUM combined with a per-reading test, $z_{i,k}^{2}>\tau_{1}$, which catches single large deviations [30].

### 4.3. Windowed $\chi^{2}$ Detector

As an alternative with bounded memory, we use the windowed statistic

(9) $$s_{i,k}=\frac{1}{W}\sum_{j=0}^{W-1}z_{i,k-j}^{2},$$

with an alarm when $s_{i,k}>\tau_{\chi }$. In the simulation, the window covers the last W=10 readings of the sensor (1 h in Experiment 1 and 4 h in Experiment 2), the statistic is evaluated once at least three readings are available, and $\tau_{\chi }$ is calibrated to the same 1% false-alarm rate. On the real-data benchmark of Section 5.7, the window is 60 samples (60 s). Unlike the single-sample $\chi^{2}$ test of [13,31], this statistic has a bounded memory of *W* readings; exponentially weighted statistics such as the multivariate exponentially weighted moving average (EWMA) considered in [31] are an alternative with fading memory. Appendix C lists all detector settings and thresholds.

### 4.4. Isolation and Rewind-and-Refilter Mitigation

Detection alone does not remove the damage already done to the state estimate: by the time an alarm is raised, the filter has absorbed some of the falsified readings. The mitigation therefore removes the compromised sensor and repairs the estimate. The estimator keeps the set S of trusted sensors (initially all) and a history of its priors $\left({\hat{x}}_{k|k-1},\Sigma_{k|k-1}\right)$, inputs and readings. When sensor *i* raises an alarm at step *k*:

1. **Isolate:** Remove *i* from S; its readings are ignored from then on. If other sensors measure the same state (redundant sensors, Section 5.4), remove them as well, because a disagreement between two sensors of the same quantity does not reveal which one is faulty or falsified.
2. **Estimate the onset:** Take ${\hat{k}}_{a}$ as the last step at which the CUSUM statistic of sensor *i* was zero, the classical change-time estimate of CUSUM [37] (for the windowed detector, the first step in the alarm window).
3. **Rewind and re-filter:** Restore the stored prior at ${\hat{k}}_{a}$ and re-run (2)–(4) up to step *k* using only the sensors in S, i.e., with $C_{S}$ and $R_{S}$.
4. **Fall back:** If S becomes empty, continue with model-only prediction (Scenario A).

The controller then continues from the repaired estimate. Isolation is permanent within a run. Section 5.1 tests whether an attacker can exploit this, and evaluates an optional re-admission rule: an isolated sensor whose innovations, still computed but not used, have a mean square below 1.5 over 20 readings is returned to S. The computational load is small. With n=7 states and m=3 sensors, one filter update with the CUSUM test needs about 3400 floating-point operations, dominated by the covariance prediction; the rewind repeats this for each stored step, and the history needs about 0.5 kB per step in double precision (53 kB for 10 h, or 11 kB if the rewind is limited to 2 h, which covers all onset estimates we observed). In our Python 3.13 implementation, on one core of a server processor, an update with detection takes 34 μs and a full rewind over 10 h takes 3.5 ms, against a sampling interval of 6 min. The detector therefore fits the edge node of the architecture in Figure 1 with a large margin; even a microcontroller sustaining a few million floating-point operations per second would complete an update in about a millisecond. The sensors themselves need no change.

### 4.5. Data-Driven Baselines

We compare the proposed detector with static and predictive data-driven detectors, all trained on attack-free operation only and calibrated to the same 1% false-alarm rate on separate attack-free runs (settings in Appendix C).

**Static detector.** An Isolation Forest [38] (300 trees) scores each reading by how unusual it is. Its features are the deviations of the readings from their targets, $y_{k}-Cx_{k}^{\mathrm{ref}}$, their means over the last five readings, their first differences, and the deviations of the control inputs from their feedforward values together with their five-reading means. It is trained on 150 attack-free runs, and its alarm rule (anomaly score above a threshold at two consecutive readings) is calibrated on 300 further runs.

**Range check.** A simpler static detector compares each reading with the mean and standard deviation of that reading at the same time step over 300 attack-free runs, and raises an alarm when any normalized deviation exceeds a threshold at two consecutive readings. It follows the time-varying normal band of each reading, like the plausibility checks common in practice. We use it to test whether the failure of static detection comes from the feedback or from the Isolation Forest itself.

**Predictive detectors.** Two one-step predictors are learned from 300 attack-free runs: a linear model (ridge regression) and a neural network (a multilayer perceptron with two hidden layers of 64 units each; the network used on HAI in Section 5.7 is a different, larger one). Each predicts the next reading of every sensor from the last five readings and inputs and the current targets. Their prediction errors, normalized on 500 further attack-free runs, feed the same CUSUM test as the proposed detector. These detectors learn the dynamics from data instead of taking them from the plant model.

## 5. Results

Unless stated otherwise, results are means over the same 100 Monte Carlo runs per configuration, with thresholds calibrated for a nominal 1% false alarms per 10-h run (effective rate about 1.5%, Section 4), and attacks start at t=3 h (replay at t=4 h). Each run draws its process and measurement noise from a seeded random generator; the evaluation seeds (1 to 100) are the same for all configurations, so that comparisons are paired, and thresholds and data-driven baselines are calibrated and trained on separate seeds. No parameter was tuned on attacked runs: the CUSUM drift is fixed at ν=0.5, every threshold follows from the false-alarm target, and the settings of the baselines (Appendix C) were fixed before evaluation. Uncertainty is reported as 95% Wilson intervals for detection probabilities and 95% paired bootstrap intervals for cost increases; Section 4 gives the uncertainty of the thresholds themselves. A full set of runs for one configuration takes about one second.

### 5.1. Impact of Attacks and Effect of the Defense

Table 3 reports the tracking cost under each attack for the three architectures and for Scenario C with detection and mitigation (C+D).

**The energy meter is the most valuable target.** With good sensors, an energy meter that under-reads by 1.0 raises the cost of Scenario C from 48.8 to 83.6, an increase of 71% (95% CI 67 to 76%): the controller believes energy is low, raises it, and the true energy level settles well above target. The same bias on the inventory or spoilage sensor leaves the cost unchanged within its uncertainty but still harms the plant, which the cost alone does not show. An under-reported inventory leads the controller to produce more, and mean spoilage rises by 6.5% (1.051 to 1.119); an under-reported spoilage index raises true spoilage by 4.5%. In Experiment 1, replay is the most damaging attack: looping four hours of old readings more than doubles the cost of Scenario C (111.0, +128%, CI 120 to 135%) and raises spoilage by 27%. Scenario C suffers even more than the raw-data Scenario B (99.9), possibly because the replayed readings are mutually consistent, so the filter has no reason to discount them.

The ranking of single-sensor targets is the same under the three weightings we tested. With equal weights on all states, or with inventory weighted like energy and spoilage, the energy bias still raises the cost (by 15% and 23%), while biases on the inventory and spoilage sensors change it by at most 2% or lower it. The magnitudes, however, depend strongly on the weights: replay, which disturbs every state, becomes far more costly (+774% with equal weights) once inventory and production carry more weight.

**IoT data bring both benefit and vulnerability.** Without attack, Scenario C is the architecture with the lowest cost (48.8 against 53.3 for A and 58.6 for B), as in [1]. Scenario A ignores the sensors and is therefore immune to all data attacks, but it gives up the benefit of IoT in normal operation. Scenario B, which trusts raw readings, is the most exposed: in Experiment 2, the energy bias raises its cost to 155.5. The Kalman filter dampens attacks but does not stop them. Denial of service is the mildest attack for Scenario C, which falls back smoothly on model predictions when readings are lost (49.7, +2%, CI −2 to 6%). In Experiment 2 the cost of Scenario C under DoS is not significantly different from the attack-free cost (−3%, paired CI −9 to 3%), and for Scenario B it is lower (−15%, CI −19 to −11%) because losing readings also removes noisy overwrites.

**Figure 2:**
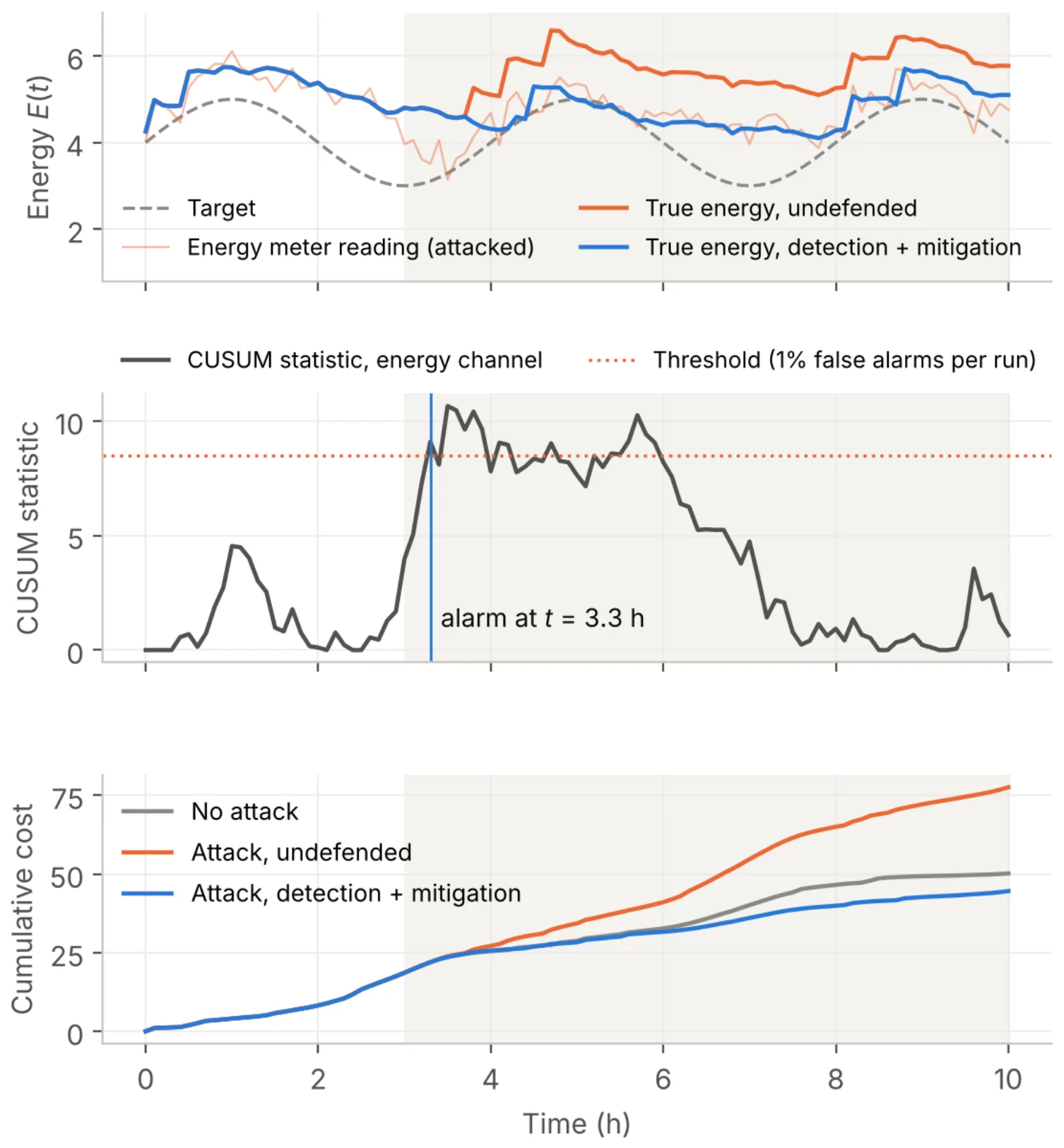
A single run of Experiment 1 with the energy meter under-reading by 1.0 from t=3 h (shaded). (**Top**): target, attacked meter reading, and true energy with and without the defense. (**Middle**): CUSUM statistic of the energy channel in the undefended run and its threshold; in the defended run, the meter is removed at the first alarm (vertical line) and its statistic is no longer used. (**Bottom**): cumulative cost without attack, under attack, and under attack with detection and mitigation. In this run the defended cost ends slightly below the attack-free cost because of the random noise of the run; over 100 runs it is 9% above.

**With good sensors, the defense removes most of the damage.** In Experiment 1, the energy bias is detected in 89% of runs, with a median delay of 0.3 h, and the mitigated cost is 53.2 (+9%, CI 4 to 15%, instead of +71%). Replay is always detected, with a median delay of 0.5 h, and the cost returns to 50.1 (+3%, CI −1 to 6%). The spoilage-index bias is detected in 98% of runs and spoilage returns to 1.055, close to its attack-free level of 1.051. Figure 2 shows a single run: the CUSUM statistic of the energy channel crosses its threshold 0.3 h after the attack starts, the meter is isolated, the estimate is repaired, and the cumulative cost then follows the attack-free curve. The defense has no measurable cost in normal operation (48.8 with and without it in Experiment 1; 63.0 against 62.9 in Experiment 2). Two attacks remain largely effective. The inventory bias is detected in only 29% of runs, because the filter absorbs an inventory offset quickly (Section 5.2). The slow energy ramp is detected in only 15% of runs, about 5 h after it starts.

**With noisy, sparse sensors, the defense rarely triggers.** In Experiment 2 only replay is reliably detected (in every run, with a median delay of 3.5 h, reducing the cost from 83.4 to 73.8). The energy bias raises the cost by 33% (CI 31 to 36%) and is detected in only 10% of runs. Section 5.4 examines why and what can be done about it.

**Permanent isolation is hard to exploit.** An attacker might trigger an alarm on purpose to remove a sensor for the rest of the run. A short, large bias of −2 on the energy meter for 0.5 h raises the undefended cost by 14%; with the defense, it is always detected, the meter is isolated for the remaining 6.5 h, and the cost rises by only 3.4%. With a bias of −3, the figures are 47% and 2.4%. The undefended damage grows faster than quadratically with the size of the bias because the energy input normally sits at its lower bound of zero (Section 3.3): a small under-reading first only moves it off the bound. The remaining inventory and spoilage readings, together with the model, estimate energy well enough, so losing the meter costs little. In particular, the mitigated cost of 53.2 under the persistent energy bias is not a return to model-only control; its closeness to the cost of Scenario A (53.3) is a coincidence. Re-admitting the meter once its innovations look normal again changes these results little (+3.6% and +2.9%), while it slightly worsens the defense against the persistent bias (+11% instead of +9%), which the rule occasionally re-admits. We therefore keep isolation permanent.

**Further checks.** Appendix D reports three further checks. The onset estimate used by the rewind is early for biases and late for slow ramps, but rewinding to the true onset would change the defended damage by at most 2 percentage points. Detection and the undefended damage after onset hardly depend on when the attack starts. A replay aligned with the targets evades detection with noisy sensors but loses most of its damage.

### 5.2. Detectability and Remaining Damage

**Figure 3:**
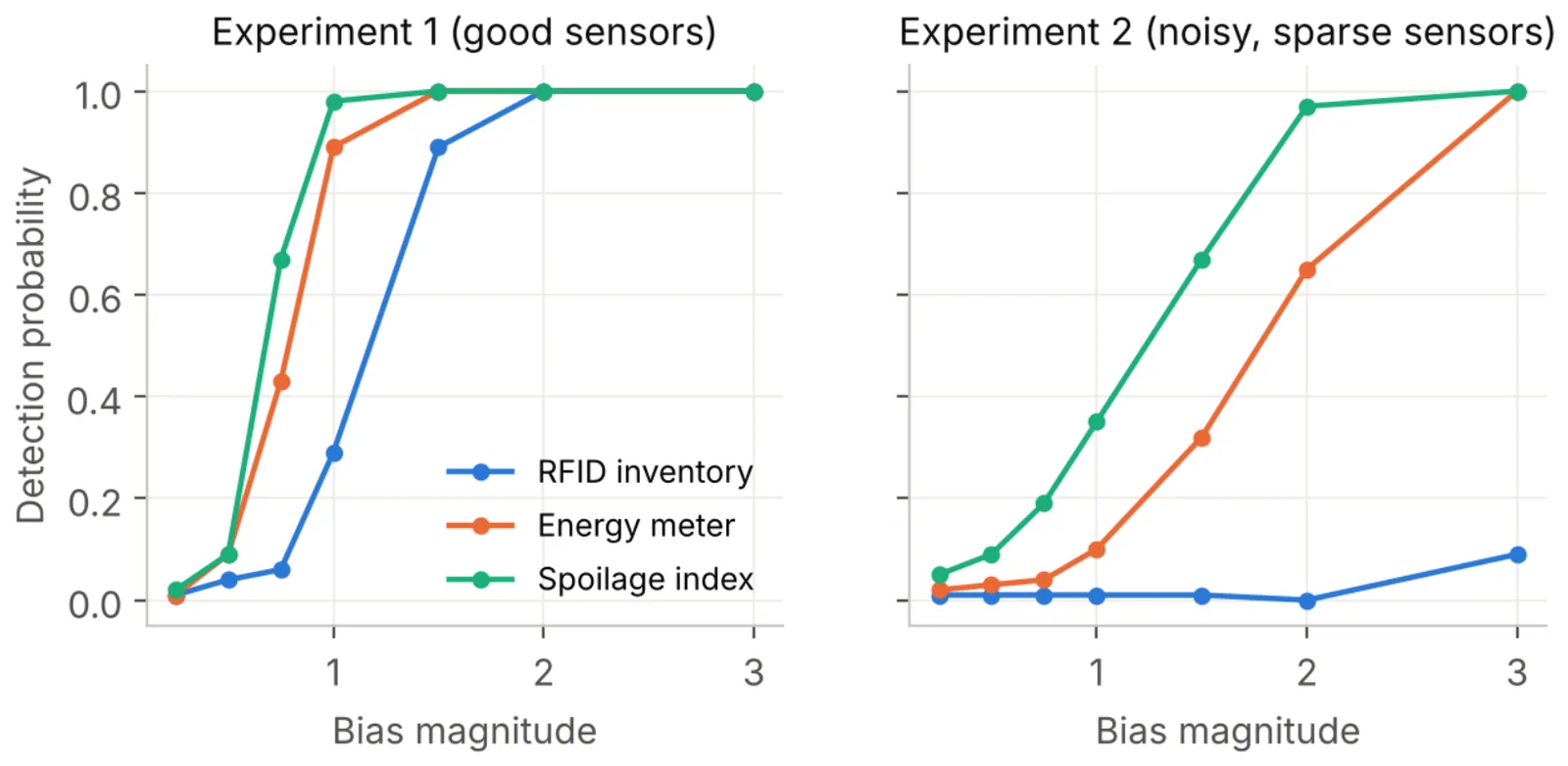
Detection probability versus bias magnitude for each sensor (100 runs per point, 1% false alarms per run).

Figure 3 shows how detection depends on the size of a bias. With good sensors, a bias of 1.0 is detected in 89 to 98% of runs on the energy and spoilage channels, but only 29% on the inventory channel; below 0.5, all three are essentially invisible. With noisy, sparse sensors, the energy channel needs a bias of 2.0 to reach 65% detection, and the inventory channel stays below 10% even at 3.0.

**Why the sensors differ.** The differences follow from the structure of the filter. For a linear plant, a sensor attack $a_{k}$ affects the innovation only through the estimation error, whose dynamics do not depend on the known control input, so the mean innovation it causes is $\bar{r}=G\left(q\right)\;a$, with *q* the forward-shift operator, with

(10) $$G\left(q\right)=I-C\;{\left(qI-A_{f}\right)}^{-1}A_{s}K,\;A_{f}=A_{s}\left(I-KC\right),$$

where $A_{s}=A_{d}^{\;n_{s}}$ is the plant over one sampling interval of $n_{s}$ steps and *K* the steady-state filter gain. This holds exactly in closed loop, even with clipped inputs, because the filter uses the same inputs as the plant. Two conclusions follow. First, no single-sensor attack is perfectly undetectable: with any one sensor *i* removed, the pair $\left(A_{s},C_{-i}\right)$ formed with the other two rows of *C* remains observable, which rules out undetectable attacks on one sensor [11]. Some attack directions are nevertheless only weakly detectable, as the transfer function shows. Second, a constant bias is visible mainly during a transient. The filter soon moves its estimate so as to explain most of the offset, and the steady-state innovation per unit bias, $G_{ii}\left(1\right)$ in standard deviations, is only 0.011 for inventory, 0.066 for energy and 0.21 for spoilage in Experiment 1 (Table 4). A persistent bias smaller than $\nu /G_{ii}\left(1\right)$ (47, 7.5 and 2.3 in Experiment 1) keeps the steady-state innovation below the CUSUM drift, so it can only be caught during the transient. The transient is weakest for inventory: its innovation decays within about half an hour and then reverses sign, and it accumulates less than a sixth of the evidence of the other two sensors over three hours. With noisy, sparse sensors the response at onset shrinks by a factor of about 4, and the evidence accumulated over three hours by a factor of 2 to 3.6. The evidence accumulated over the first three hours reproduces the simulated ranking of the sensors in both experiments (Table 4). Feeding the noise-free mean innovation to the CUSUM gives conservative alarm predictions, because noise sometimes carries a borderline statistic over the threshold: it predicts no alarm for the inventory bias in Experiment 1 and the energy bias in Experiment 2, which are detected in 29% and 65% of runs. Across sensors, the weakest direction is almost entirely the inventory sensor (the smallest singular value of the normalized G(1) belongs to the direction (−0.98,0.07,−0.16)), which is why the detector-aware attacker below does most harm to spoilage through that sensor.

**Figure 4:**
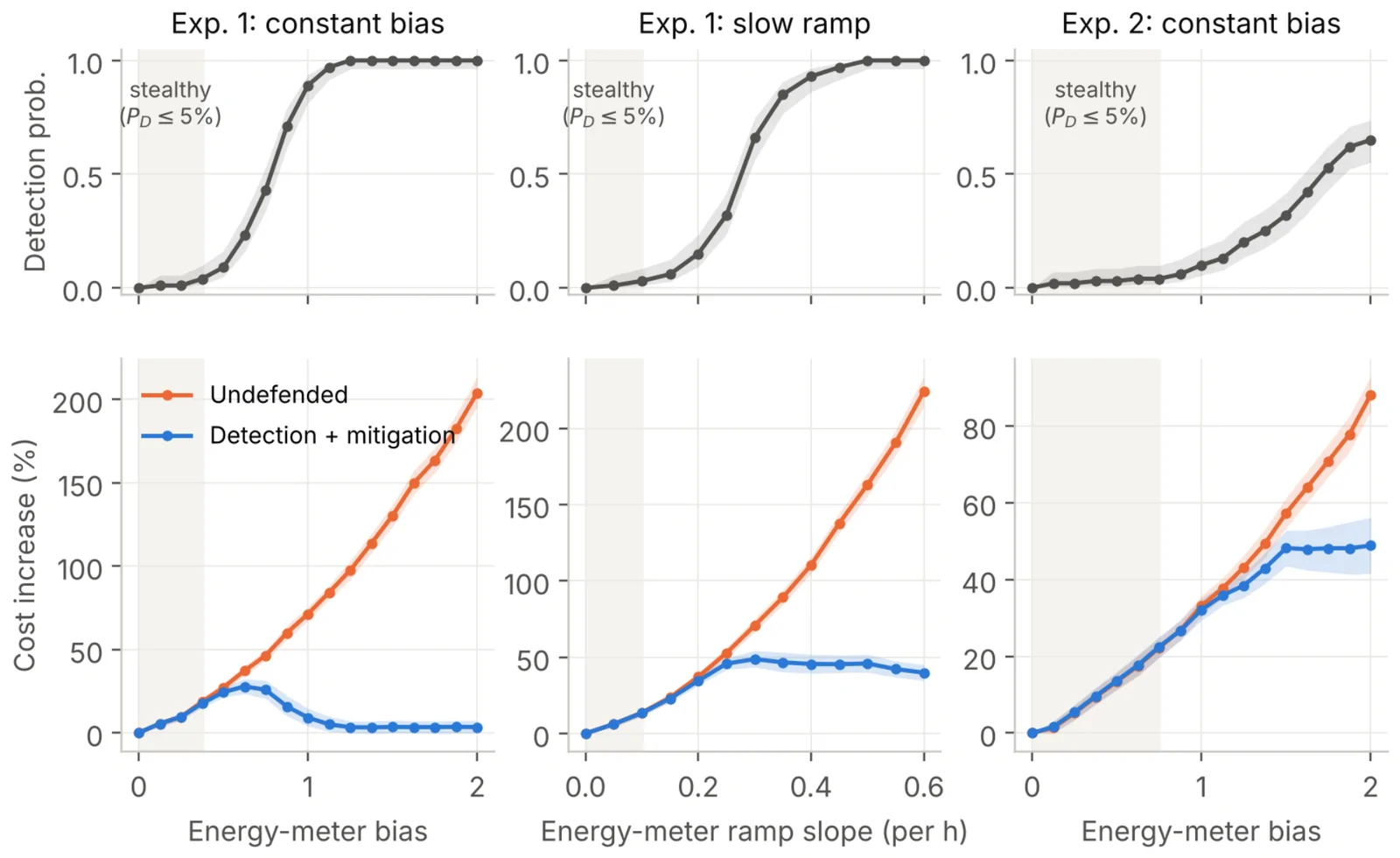
Detection probability (**top**) and cost increase relative to the attack-free cost (**bottom**) versus the strength of energy-meter attacks, without and with detection and mitigation, with 95% Wilson (**top**) and paired bootstrap (**bottom**) intervals. Vertical scales differ between the lower panels. Shaded: attacks detected in at most 5% of runs.

**Fixed-shape attacks.** Small attacks go unnoticed, but they still cause damage. Figure 4 plots detection probability and cost increase against the strength of energy-meter attacks. In Experiment 1, the largest bias detected in at most 5% of runs (b=0.375) raises the cost by 19% (CI 9 to 28%), and the largest such ramp (0.1 per hour) by 14% (CI 6 to 26%); these are the stealthy damages $D_{0.05}$ of (6). In Experiment 2, the stealthy damage for a bias is 22% (CI 5 to 33%, at b=0.75). The wide intervals reflect how sensitive the largest undetected attack is to the estimated detection probabilities.

With the defense active, damage is no longer monotone in attack size. Large attacks are caught quickly and cost little (+3% for biases of 1.25 and above in Experiment 1). The attacker’s best fixed-shape strategy is therefore an intermediate attack, large enough to cause harm but small enough to be caught late or not at all. The worst-case defended damage is 28% for a bias (CI 24 to 32%, at b=0.625, detected in 23% of runs) and 49% for a ramp (CI 45 to 54%, at 0.3 per hour, detected in 66% of runs but late). In Experiment 2 it is at least 49% (CI 45 to 56%), reached at the largest bias tested (50% in Section 5.4, where the threshold is recalibrated for each sensing design).

**Detector-aware attacks.** An attacker who knows the filter and the detector can do much more (Table 5). Keeping the CUSUM statistic of the energy meter just below its threshold, it raises the cost by 121% in Experiment 1 and 93% in Experiment 2 and is flagged no more often than an attack-free run. For this comparison, which needs the thresholds of CUSUM with and without a per-reading test estimated on the same runs, all thresholds were recalibrated on a fresh set of 1000 attack-free runs; this gives τ=9.0 and 6.7, just above the bootstrap intervals of Section 4, and with the thresholds of Table 3 the figures are 116% and 89%. Watching the other sensors as well (the full-bank attacker) gives exactly the same result for the energy meter, because an energy offset is absorbed by the energy and machine-performance estimates and barely moves the inventory and spoilage innovations. Adding a per-reading test does not help either: it only caps the attacker’s first push, which the CUSUM limit constrains later anyway, and the higher CUSUM threshold needed to keep the same false-alarm budget gives the attacker slightly more latitude (+124% and +97%). Against the windowed $\chi^{2}$ detector, the same strategy raises the cost by 361% and 197% with a window of 10 readings, and by 541% and 201% with 5 readings, the window that works best against fixed-shape biases. The stateful CUSUM thus limits a greedy stealthy attacker two to four and a half times better than the bounded-memory statistic, in line with the comparisons of [13,31], but neither prevents large undetected damage with a single energy meter.

The detector-aware attack on the inventory sensor raises spoilage by 22 to 31% and, unlike a fixed-shape inventory bias, also raises the cost (by 30 to 31%). Its alarms (25% of runs in Experiment 1, 10% in Experiment 2) are never raised on the inventory sensor, which the attacker keeps below threshold, but on the energy or spoilage sensor, whose innovations respond to the corrupted estimate; the defense then isolates an uncompromised sensor. Because these alarms remove a truthful sensor while the attack continues, the defense does not reduce the damage at all (Table 5); a remedy would be to test, after an alarm, which sensor’s exclusion restores white innovations, in the spirit of secure state estimation [28,39]. The full-bank attacker avoids some of these alarms in Experiment 2 (5% instead of 10%) at the price of a smaller cost increase (+20% instead of +31%), while spoilage still rises by 29%. Attacks on the spoilage index have no effect on cost, because no input acts strongly on spoilage. The damage from fixed-shape attacks reported above is therefore a lower bound; the detector-aware results show what an attacker who knows the detector, but plans greedily, can reach.

### 5.3. Static Versus Predictive Detection in Closed Loop

**Figure 5:**
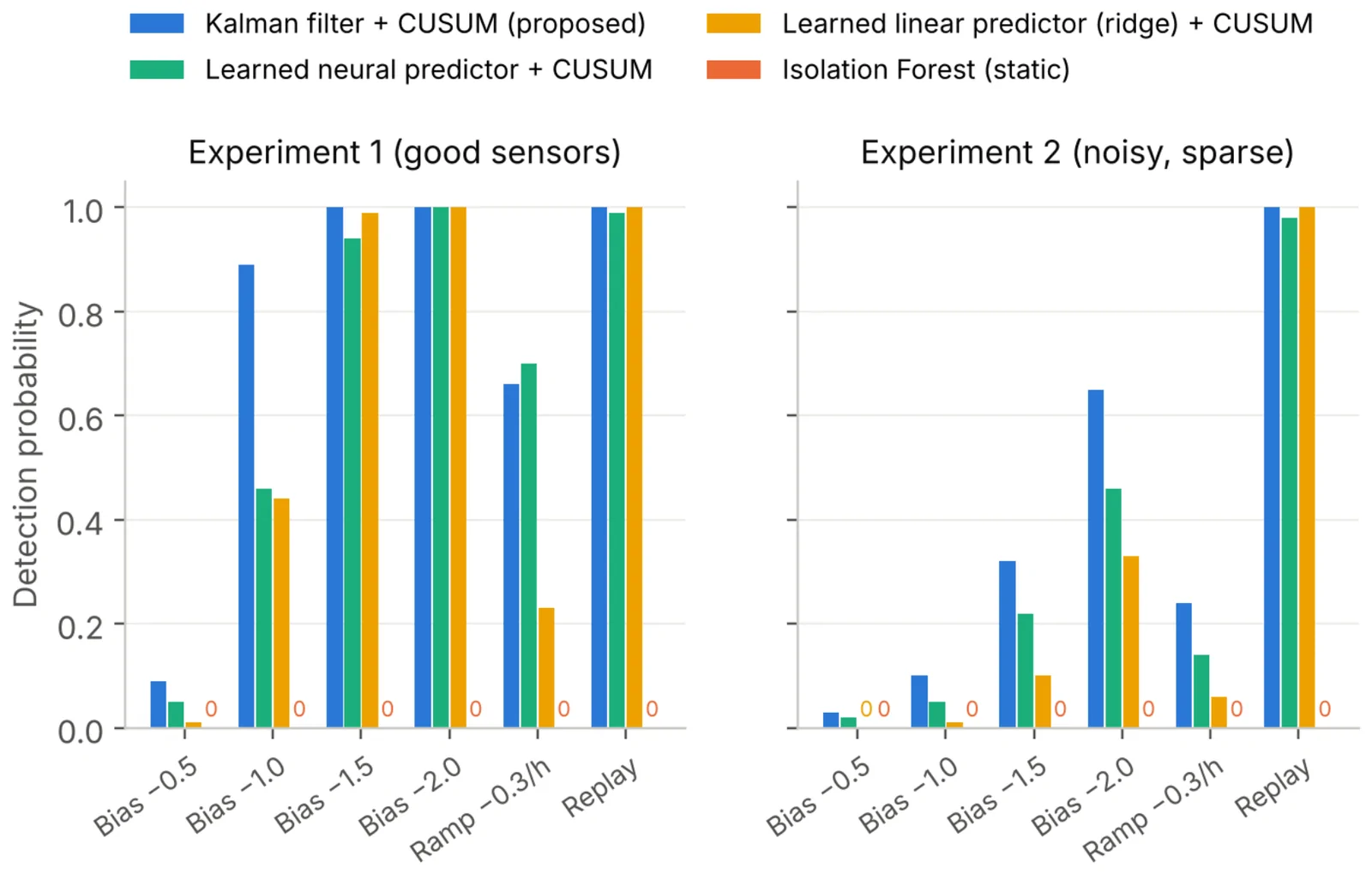
Detection probability at the same false-alarm rate (1% per run) of the proposed detector (Kalman filter with CUSUM), two data-driven predictive detectors (learned neural and linear one-step predictors with CUSUM), and a static data-driven detector (Isolation Forest), for energy-meter biases and ramps and for replay (100 runs per bar).

The Isolation Forest, calibrated to the same false-alarm rate, detected none of the attacks in either experiment (Figure 5), including energy biases of 2.0, about nine measurement standard deviations in Experiment 1, and replay. The detectors that predict the dynamics from data behave very differently. With good sensors, the learned neural predictor detects a bias of 1.5 or more in at least 94% of runs, a ramp of 0.3 per hour in 70%, and replay in 99%; the learned linear predictor does as well except on ramps. They are weaker than the Kalman-filter detector on moderate biases (46% and 44% against 89% at a bias of 1.0), because they must learn from noisy data what the Kalman filter takes from the plant model, and they are weaker still with noisy, sparse sensors.

The reason is feedback. The controller acts on the falsified reading and steers it back towards its target, so the reading stays inside the range seen in normal operation while the true state drifts away. The control inputs do move, but by less than their normal variation along the time-varying targets. A replayed reading is, by construction, a sample of normal operation. Each reading therefore appears normal on its own; what is abnormal is the inconsistency between successive readings, inputs and the process dynamics. Any detector that predicts the next reading sees this inconsistency, whether its predictor is a first-principles model or is learned from data; a detector that only models the distribution of individual readings does not.

Two checks confirm this explanation and show that it is not an artifact of the Isolation Forest. First, the range check (Section 4.5) detects an energy bias of 1.0 in 92% of runs and a ramp of 0.3 per hour in all runs when the falsified readings are applied after the run, so that the controller never reacts to them (open loop). In closed loop it detects them in only 14% and 3% of runs: two hours after the attack starts, the falsified reading lies on average 0.3 standard deviations of its normal band from normal, against 2.8 in open loop. It catches large biases in closed loop (91% of runs for a bias of 2.0) during the transient, before the controller has compensated. The Kalman-filter detector finds the bias of 1.0 in 89% of runs and the ramp of 0.3 per hour in 66%. Second, the Isolation Forest detects none of these biases even in open loop, and giving it the last five readings and inputs raises its detection only to 21% for a bias of 2.0 and 27% for replay. An Isolation Forest is thus a weak detector of a shift in one feature among many, and the range check is the fairer static baseline. With either, the conclusion is the same: in closed loop, detectors that judge readings one at a time miss the moderate and slow attacks that a predictive detector finds.

Table 6 summarizes the comparison with the measures used in intrusion detection. All detectors work at the same false-alarm rate, so the run-level precision is high whenever an attack is detected at all, and the F1 score follows recall. The Kalman-filter detector has the highest or equal-highest F1 score on every attack except the ramp in Experiment 1, where the learned neural predictor is slightly better (0.82 against 0.79). Precision depends on how rare attacks are: if only one run in a hundred were attacked, a detector with 89% recall and a 1% false-alarm rate would have a precision of about 47%, so that every second alarm would be false. Section 5.6 examines what such false alarms cost.

### 5.4. Sensing Design for Security

**Figure 6:**
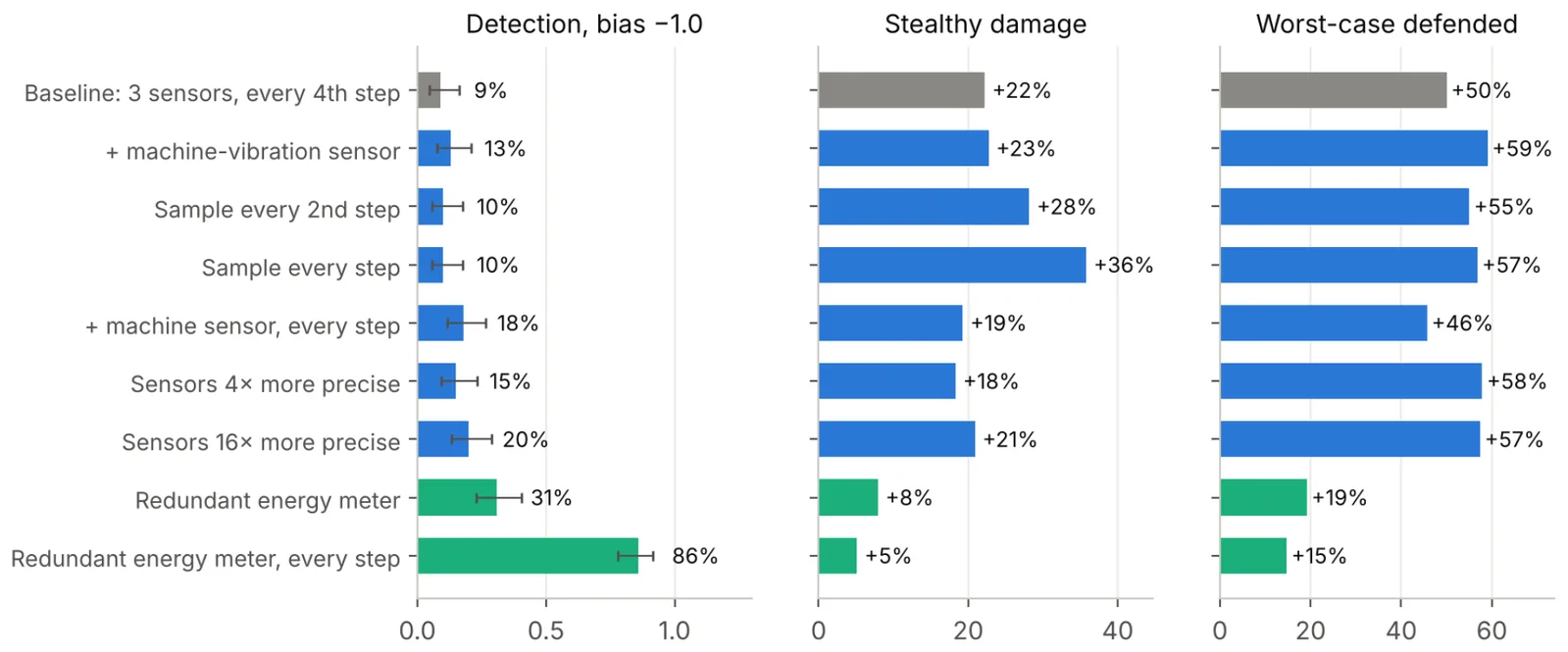
Effect of the sensing design on the detection of a −1.0 energy bias, the stealthy damage, and the worst-case defended damage, under the process noise of Experiment 2 (100 runs per design).

Why is the defense so weak in Experiment 2, and can better sensing fix it? Table 7 and Figure 6 compare nine sensing designs under the process noise of Experiment 2. In each design the detector is recalibrated to 1% false alarms, which is why the baseline values differ slightly from those of Table 3 and Figure 5 (for example, 9% instead of 10% detection of the energy bias, and 22% instead of 24% for the ramp of 0.3 per hour). The machine-vibration sensor measures machine performance with the same noise variance as the energy meter (0.8); “4× more precise” means that all sensor noise variances are divided by 4.

Improvements in sampling and precision help little. Adding a machine-vibration sensor, sampling every step instead of every fourth, or making the sensors 4 or 16 times more precise leaves detection of the energy bias between 10% and 20% and the stealthy damage between 18% and 36%, differences that are mostly within the uncertainty of these estimates (Section 5.2). More frequent or more precise readings even make undetected attacks more harmful (a bias of 1.0 costs +33% in the baseline design but +47 to +55% with precise sensors), because the filter trusts the sensors more and follows the attacker more closely. The underlying reason is that, with large process noise, a biased reading is statistically almost indistinguishable from a genuine change in energy. A model of the dynamics, however accurate its data, cannot tell the two apart.

A second energy meter can, because a disagreement between two measurements of the same quantity is a direct inconsistency. This is the principle of hardware redundancy in fault detection [24] and of secure estimation with a compromised subset of sensors [28]; here we quantify its value for this plant. The second meter lowers the stealthy damage from 22% to 8% and the worst-case defended damage from 50% to 19%. Sampled every step, it raises detection of the −1.0 bias from 9% to 86% and of the 0.3-per-h ramp from 22% to 99%. A fairer comparison for this row is the single meter sampled every step, whose stealthy damage is 36% against 5%. Part of the gain comes from the filter fusing two readings, which halves the effect of one falsified meter even before detection. At the same sampling rate, the redundant meter also improves normal operation slightly ($J_{0}=60.4$ against 62.9).

Table 8 tests the redundant design against stronger attackers. Against the own-channel detector-aware attacker on one meter, redundancy cuts the damage from 93% to 25% (every fourth step) and from 169% to 20% (every step). This attacker is now detected in 21% and 48% of runs, always through the second meter, whose readings disagree with the estimate pulled away by the falsified one. The full-bank attacker, which also watches the second meter, must keep that disagreement small. With its look-ahead alone it gains no advantage (25% and 20%, detected in 20% and 45% of runs), because noise on the second meter still triggers alarms. If it also keeps that meter’s predicted normalized innovation below $\bar{z}=0.4$, it is detected in only 3% and 0% of runs but raises the cost by only 15% and 10%; with any tighter bound no damaging push remains, and the attacker’s best choice lowers the cost. For a greedy detector-aware attacker on one meter, redundancy therefore limits the undetected damage to about 15%, against 93% with a single meter. Isolating both meters on an alarm matters: with per-sensor isolation, the defense sometimes removes the uncompromised meter, and the defended cost of the own-channel attack rises to 31% and 45%. Redundancy offers no protection if the attacker can falsify both meters consistently, as a gateway or edge attacker can. It then increases the damage: the filter trusts two agreeing meters more than one, and the full-bank attacker on both meters raises the cost by 167% and 297% undetected, against 93% and 169% for a single meter. The benefit of redundancy therefore depends on the two meters reporting over independent paths, for example through separate gateways, or on authenticating each meter’s readings end to end.

**Benefit and cost of the sensing designs.** Table 9 brings together, for each design, what it requires and what it buys. The designs differ in hardware (an additional sensor or more precise sensors), in communication (readings per hour, which drives the energy use of battery-powered LoRaWAN nodes), and in protection against three kinds of attackers. Only the redundant meter lowers the damage of all three, and it does so for one additional meter and, at the original sampling rate, one third more readings. Its value rests on the assumption that the two meters cannot be falsified together; if they can, the same design raises the undetected damage from 93% to 167%. Because the cost *J* is not monetary, the economic decision has to be made with a plant’s own figures. A simple rule follows from the table: the redundant meter pays off when the expected loss it avoids, the probability of a falsification attack over the planning period times the monetary value of the damage avoided, exceeds the cost of buying, installing and maintaining the meter and its independent communication path. Faster sampling and more precise sensors cost communication energy or hardware without lowering the stealthy damage, and are not worth buying for security alone.

### 5.5. Model Mismatch and Choice of Detector

In the simulations above, the Kalman filter uses the exact plant model, so its innovations are white and CUSUM is near-optimal for persistent shifts. Real plants differ from their models. We tested two kinds of mismatch, keeping the nominal model in the filter and the controller (Table 10). For parameter mismatch, every nonzero entry of the plant’s *A* and *B* matrices was perturbed by a random relative error with standard deviation ϵ. We drew 20 such plants for each ϵ, calibrated both detectors on 300 attack-free runs of each plant, as an operator would calibrate on the plant at hand, and evaluated them on 50 further runs of the same plant. For noise mismatch, the true process or measurement noise variance was doubled or halved, and the detectors were recalibrated on 500 attack-free runs.

Thresholds calibrated on the model do not transfer to a mismatched plant: with parameter errors of 10% and 20%, 6% and 41% of attack-free runs raise a CUSUM alarm in Experiment 1 (windowed statistic: 1% and 7%), and the thresholds recalibrated on individual plants range from 9 to 57 at ϵ=0.2. Calibrated on the plant itself, the defense keeps its false-alarm budget and rarely isolates a sensor without attack (in at most 1.4% of attack-free runs, at a cost below 1.2%), but it loses power. In Experiment 1 CUSUM detects the energy bias in 84% of runs with an exact model (rather than 89%, because each threshold is now estimated from only 300 runs), 58% at ϵ=0.1 and 24% at ϵ=0.2, with a wide spread between plants (0 to 63% at ϵ=0.2), and the defended cost increase grows from 11% to 32% and 53%, against 66 to 72% without defense. Replay is still detected in 99% of runs, but the damage left by the defense grows from 4% to 12% and 46%, because the estimate of repairs is built on a less accurate model. The windowed statistic degrades more slowly: it overtakes CUSUM on the energy bias from ϵ=0.1, and at ϵ=0.2 it leaves 34% and 26% of damage from the bias and replay, against 53% and 46% for CUSUM. With noisy, sparse sensors, the defense was already weak against the bias, and at ϵ=0.2 mismatch removes its effect on replay as well.

Noise mismatch separates the two detectors. When the process noise is underestimated, the innovations acquire persistent structure that CUSUM accumulates: its nominal threshold gives 35% false alarms, and after recalibration it detects the energy bias in only 13% of runs, against 51% for the windowed statistic. When the measurement noise is underestimated, the innovations stay white but are too large, and the windowed statistic suffers instead (78% false alarms with nominal thresholds, 8% detection after recalibration, against 64% for CUSUM). Overestimated noise keeps both detectors usable: CUSUM gains most when the process noise is overestimated (100% detection of the bias) and the windowed statistic when the measurement noise is (99%). The window length of the windowed statistic matters as well (Appendix D).

The choice of detector should therefore follow the quality of the model, which can be checked before deployment on held-out normal data: CUSUM when the normalized innovations are white with unit variance, and a bounded-memory statistic when they show persistent structure. In every case the thresholds should be calibrated on the plant rather than on the model. On HAI (Section 5.7), where the model is identified from data, the residuals are nearly white but heavy-tailed, and CUSUM with the drift of the simulation keeps its alarm on during 51% of attack-free time.

### 5.6. Robustness to Operating Conditions and Attacker Knowledge

This subsection varies one condition at a time around the nominal settings: the detector threshold, the measurement noise, packet loss, the timing of the attack, and the attacker’s knowledge. Model mismatch is treated in Section 5.5.

**Figure 7:**
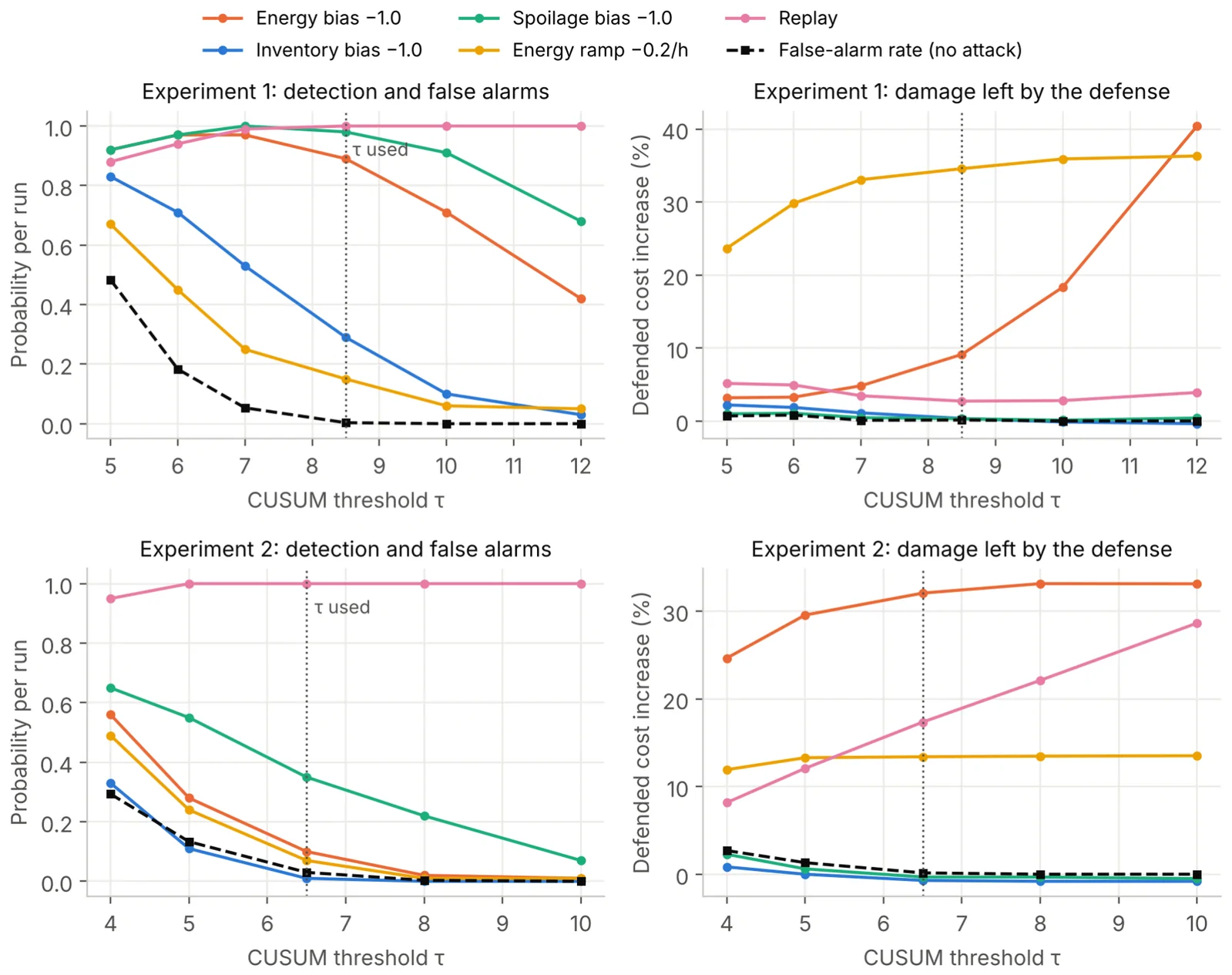
Effect of the CUSUM threshold. Left: detection probability for five attacks and false-alarm rate per 10-h run (300 attack-free runs; at the thresholds used in the paper, the 7000 runs of Section 4 give the more precise rates of 1.5% and 1.6%). Right: cost increase that remains with the defense active; the black line is the attack-free cost with the defense, i.e., the cost of false alarms. Dotted lines: thresholds used in the paper.

**Detector threshold, cost of false alarms and of missed detections.** The 1% false-alarm rate used so far is a design choice. Figure 7 shows what happens when the threshold is moved. Lowering it from 8.5 to 7 in Experiment 1 raises the false-alarm rate from about 1.5% to 5% of runs and detection of the inventory bias from 29% to 53% and of the slow ramp from 15% to 25%, and it halves the damage left by the energy bias (from 9% to 5%). A false alarm is cheap in this architecture, because it only removes one sensor: in attack-free runs in which the defense isolated a sensor, the cost rose by 1 to 4% in Experiment 1 and by 5 to 13% in Experiment 2, where each remaining sensor matters more. False alarms were spread over all three sensors (for example, 34, 55 and 56 first alarms on the inventory, energy and spoilage sensors at τ=5 in Experiment 1), and losing the spoilage index cost least. A missed detection is far more expensive and depends on the attack: an undetected energy bias costs 62 to 75% in Experiment 1 and 33% in Experiment 2, an undetected ramp 36% and 13%, while undetected biases on the inventory and spoilage sensors barely change the cost but raise spoilage (Table 3). Even at a 48% false-alarm rate per run (τ=5), the defense raises the attack-free cost by only 0.7% in Experiment 1 and, at 29% (τ=4), by 2.7% in Experiment 2. The practical limits on lowering the threshold are therefore the operator’s workload and the permanent loss of sensors over long operation (Section 4), not the cost of the false alarms themselves.

**Measurement noise.** With the process noise of Experiment 1 and the measurement noise variances scaled by 0.25, 1, 4 and 16, and the threshold recalibrated at each level on 3000 attack-free runs to the false-alarm rate of the nominal threshold, detection of an energy bias of fixed size 1.0 falls from 99% to 89%, 56% and 25%, and the damage left by the defense rises from 5% to 9%, 27% and 38%. The decline reflects the size of the bias relative to the noise: a bias of 1.0 is about 4.5 noise standard deviations of the energy meter at the nominal level and 1.1 at the highest level, close to the meter of Experiment 2. A bias that grows with the noise standard deviation (2.0 and 4.0 at the two highest levels) is detected in every run, and the defense limits its damage to 3% or less. Replay remains detected in every run, and the slow ramp stays hard to detect at every level (12 to 15%).

**Packet loss.** Random loss of 20% and 50% of all readings, combined with the attacks, does not raise the false-alarm rate, but it slows detection. In Experiment 1 detection of the energy bias falls from 89% to 77% and 52%, and the damage left by the defense rises from 9% to 18% and 36%; replay is still always detected, with the remaining damage rising from 3% to 12%. In Experiment 2, 50% loss lowers detection of replay to 83%.

**Intermittent attacks.** Table 11 switches the energy bias on and off. Intermittency does not help the attacker against CUSUM unless the bias is on only briefly: every switch causes a new transient in the innovations, and a bias on for half of each hour is detected in 99% of runs. Short pulses are the exception. A bias on for 6 min of every hour (duty cycle 0.1) is detected by CUSUM in only 2% of runs, because its statistic decays between pulses, but it also does little damage (+12%), and a per-reading test or the windowed statistic catches it (93% and 69%). A pulse shorter than the sampling interval may be missed altogether: with readings every 0.4 h in Experiment 2, 6-min pulses often fall between readings and have no effect at all. Combining CUSUM with a per-reading test is therefore the simple adjustment for intermittent attacks.

**Imperfect attacker knowledge.** Table 12 replaces the fully informed detector-aware attacker on the energy meter with one that knows the model only up to relative errors $\epsilon_{a}$, does not see the detector’s state before the attack, and assumes a threshold fτ. Without the detector’s state, even an exact model is not enough: an attacker who assumes the true threshold is caught in 26% (Experiment 1) and 29% (Experiment 2) of runs, because the statistic it cannot see has already accumulated noise. A conservative attacker (f=0.8) stays undetected in 94% of runs and still raises the cost by 111% and 79%. Model errors make the attack visible: with errors of 5 to 20%, the conservative attacker is detected in 36 to 57% of runs in Experiment 1, and the defense then reduces its damage from 104% to between 85% and 49%. An attacker who overestimates the threshold by 20% is always detected at once, and the defense then limits its damage to 2 to 4%. The fully informed attacker of Table 5 is therefore a demanding upper bound; a realistic attacker must trade damage against a substantial risk of detection.

### 5.7. Validation on a Real Industrial Testbed

The simulation cannot show that residual detection works on a real plant with real attacks. We therefore test the detection approach, though not the perishable-goods model and not the mitigation, on the HAI 21.03 dataset [15], the second release of the HAI testbed [40].

**Data.** HAI is a hardware-in-the-loop testbed in which a boiler (Emerson Ovation DCS; Emerson Electric Co., St. Louis, MO, USA), a turbine (GE Mark VIe; General Electric, Boston, MA, USA) and a water-treatment process (Siemens S7; Siemens AG, Munich, Germany) are coupled through a real-time simulator; signals are recorded at 1 Hz. It provides 256 h of normal operation for training and 112 h of test data with labeled attacks. We use the boiler process, whose pressure, level, flow and temperature loops are the closest analogue to the energy subsystem of our plant: like the energy and storage loops of a perishable-goods plant, they are thermal and hydraulic processes under feedback control, sampled by networked sensors, whose readings are pulled back to their setpoints by the controller. The test data contain 34 attacks on it. The attacks alter setpoints or valve commands; in 16 of them the attacker also holds the affected sensor at its previous value so that the operator display looks normal, and five are short, repeated attacks. These attacks act partly on the controller side rather than only on sensor data, so the benchmark tests the detection of inconsistencies between signals rather than the exact threat model of Section 3.2. The results transfer to the application with three restrictions: HAI has no inventory, perishability or spoilage dynamics; its sampling is much faster (1 s against 6 to 24 min) and its sensors are wired rather than wireless; and its model is identified from data rather than taken from a first-principles description. What carries over is the question that matters here: whether a detector that predicts the dynamics keeps its advantage over static detectors on a real controlled process with real attacks.

**Method.** Without a first-principles model, we identify a linear predictor from normal data only: an autoregressive model with exogenous inputs (ARX; five lags, ridge least squares) predicts each of 22 boiler signals (5 valve commands and 17 sensors) one second ahead from the values of all 30 boiler signals one to five seconds earlier. Its prediction errors play the role of the Kalman innovations. They are normalized by their standard deviations on held-out normal data (the last 30% of the training period), and the windowed $\chi^{2}$ statistic (9) is computed over 60 s; an alarm is raised when its maximum over the 22 signals exceeds a threshold fixed in advance on the same held-out data for one false alarm per day. The lag order, ridge penalty and window length were fixed before looking at the test data and were not tuned. We also train a learned nonlinear predictor on the same inputs and targets, a multilayer perceptron with hidden layers of 128 and 64 units, larger than the network of Section 4.5 because it predicts 22 signals from 150 inputs. As a recent detector of a different kind, we add a windowed autoencoder in the spirit of USAD [21]: a network with hidden layers of 128, 32 and 128 units that reconstructs 60-s windows of all 30 standardized signals (sampled every 5 s), whose reconstruction error serves as the anomaly score. For CUSUM on the same residuals, we tried drifts ν of 0.5, 1, 2, 4 and 8, each with its threshold fixed on the held-out data; we report ν=0.5, the value used in simulation, and ν=8, the best of this grid on the test data, which is therefore an optimistic result. The Isolation Forest baseline uses the standardized signals and their 60-s means, and its score is averaged over 60 s so that its alarm rule matches that of the $\chi^{2}$ detector; its threshold is also fixed on the held-out data. Because Section 5.3 shows that an Isolation Forest is a weak detector of a shift in one signal among many, we add a static range check: the 60-s mean of each standardized signal, with an alarm when any of them leaves a band fixed on the held-out data for one false alarm per day. Attack segments less than 2 min apart are merged into one attack, which also covers the five short, repeated attacks. An attack counts as detected if an alarm occurs between its start and 60 s after its end. Because an alarm that is already on when an attack starts would then count, we also apply a stricter rule under which the alarm must switch on within that interval. False alarms are counted on the attack-free parts of the test data, excluding the 60 s before and the 10 min after each attack so that the recovery of the process is not counted, in two ways: as alarm episodes per day (alarms less than 5 min apart form one episode) and as the share of time with the alarm on. Because the event-level count is lenient, we also report the enhanced time-series aware precision and recall (eTaPR) scores recommended for HAI [22]. All detectors are trained on the same normal data, use the same 60-s alarm window, have their thresholds fixed on the same held-out data for the same false-alarm rate, and are scored with the same rules, so that the comparison is like for like. We do not reproduce the detectors published for HAI 21.03, including those of the anomaly-detection contest held on it [15]: they use all four subsystems and their own thresholds and scoring, whereas our aim is narrower, namely to test under one alarm rule fixed in advance whether a predictive detector keeps its advantage over static and reconstruction-based ones on real plant data. Appendix E lists the attacks. Our eTaPR scores are given to help place the results, but they cover the boiler attacks only and are not directly comparable with whole-testbed scores.

**Figure 8:**
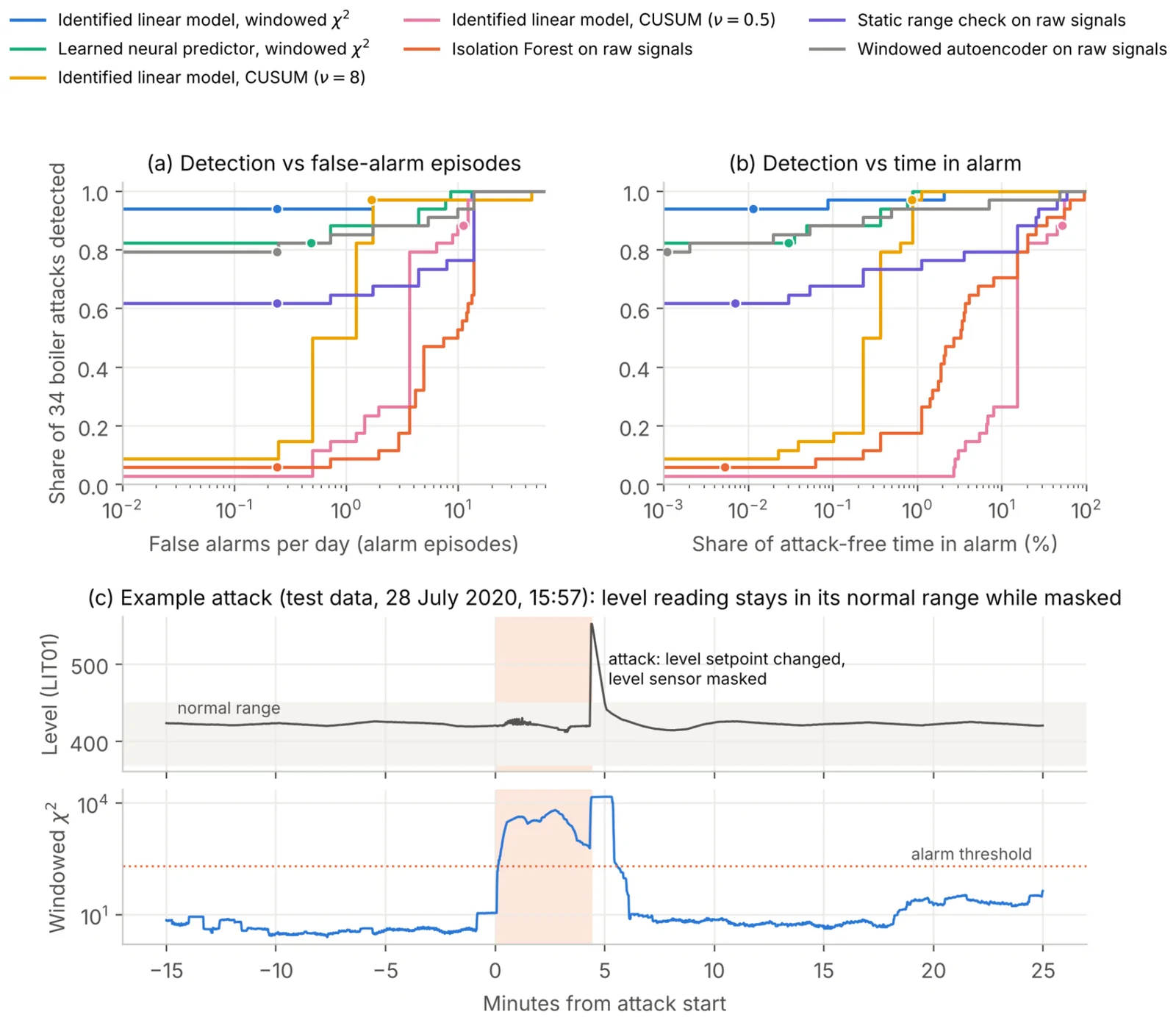
HAI 21.03 boiler. (**a**) Share of the 34 attacks detected versus false-alarm episodes per day on the attack-free test data, over all thresholds; dots mark the thresholds fixed in advance on validation data. (**b**) The same against the share of attack-free time with the alarm on. (**c**) Attack of 28 July 2020, 15:57: the level setpoint is changed and the level sensor masked. The recorded level stays inside its normal range during the attack (the jump at the end is the true level reappearing when masking stops), while the windowed $\chi^{2}$ statistic of the ARX residuals rises by two orders of magnitude within 5 s of the labeled start; a small rise about 1 min earlier stays far below the threshold. CUSUM curves use the residuals of the same identified model.

**Results.** The ARX residual detector found 32 of the 34 attacks, with a median delay of 6 s, 0.24 false-alarm episodes per day, and its alarm on during 0.01% of attack-free time (Table 13). It performed equally well on attacks with masked sensors (15 of 16) and without (17 of 18), and its eTaPR F1 score changes little when the recall criterion is made stricter (0.59 to 0.57). The learned neural predictor found 28 attacks at 0.48 false alarms per day, confirming that a data-driven detector works when it predicts the dynamics. The windowed autoencoder found 27 attacks at the same false-alarm rate, 15 of the 16 masked ones but only 12 of the 18 unmasked ones, with a median delay of 32 s; its alarms are the most precise (eTaP of 0.78) but cover less of each attack (eTaR of 0.40) than those of the ARX detector. Reconstructing a window of normal behavior captures part of the dynamics, so it sits between the predictive and the static detectors. The static range check found 21 attacks at the same false-alarm rate, with a median delay of 51 s. Its misses follow the mechanism found in simulation: it found 13 of the 16 attacks in which a sensor was masked, where the hidden process eventually drives other signals out of range, but only 8 of the 18 unmasked attacks, which the boiler’s own control loops largely compensate. The Isolation Forest, with the same alarm rule and a threshold also fixed on validation data, found 2 attacks; that it produced the same single false-alarm episode on the test data as the ARX detector is a coincidence. Figure 8 shows that these gaps hold at every threshold, whichever way false alarms are counted: the range check stays below the predictive detectors at every false-alarm rate, and the Isolation Forest needs about 10 false-alarm episodes per day, or its alarm on during several percent of the time, to detect half of the attacks. Under the stricter onset rule, the ARX detector finds 27 attacks, the neural predictor 26, CUSUM with ν=8 28 and with ν=0.5 13, and the Isolation Forest 2. The example in panel (c) illustrates the masking effect found in simulation. During the attack the level reading stays within its normal range, so a detector looking at values sees nothing unusual, while the inconsistency between the level reading and the valve commands is clearly visible to the predictive model.

The two missed attacks illustrate the limits of the approach. One (10 July, 05:29, a manipulated pressure-valve command with the pressure sensor masked) raised the statistic to 81% of the threshold, a near miss. The other (13 July, 23:55) changed the pressure setpoint while hiding the change from the operator. The plant then followed the new setpoint exactly as it would follow a legitimate one, so the recorded data remain consistent with the process dynamics. No detector based on a process model can flag such an attack; it can only be caught by checking setpoint changes against authorized operator actions.

The residuals of the identified model behave differently from the simulated innovations. They are nearly uncorrelated (median lag-one autocorrelation −0.01 on validation data and −0.03 on attack-free test data) and keep their scale on the test data (standard deviations within 8% of one), but they are heavy-tailed and come in bursts: the 60-s statistic, whose median would be about 1 for Gaussian white residuals, has a median of 2.8 and a 99.9th percentile of 146 on attack-free test data, and the threshold fixed for one false alarm per day is 201. The detector therefore works on HAI because the attacks produce residuals far larger than these bursts, not because it resolves shifts of one standard deviation as in simulation. CUSUM accumulates the bursts, which is why it needs a very large drift here.

The identified models need no labeled attacks. The ARX model trains in seconds and costs one matrix–vector product per sample to run; the neural predictor trained in about two minutes.

## 6. Discussion

**Main findings.** The results support five findings about this class of systems. (i) The damage an attack causes depends on which sensor it targets, and the ranking can be computed in advance from the plant model. (ii) In closed loop, detectors that judge readings one at a time miss moderate and slow attacks, because feedback keeps falsified readings in their normal range; predictive detectors, model-based or learned, do not have this weakness. (iii) A detector built from the existing Kalman filter removes most of the damage of attacks by an adversary who does not know the detector, given accurate sensors and an accurate model, and its power falls with sensor noise, packet loss and model error. (iv) A fully informed attacker can cause large undetected damage, but errors in its model make the same strategy detectable; only an attacker with an accurate model that stays well below the threshold remains hidden. (v) Better or faster sensing does not reduce the undetected damage, whereas a redundant sensor on an independent path does.

**Implications for practice.** Four lessons carry over to perishable-goods producers that adopt IoT-enabled control. First, not all sensors are equal targets. In our plant the energy meter is the one whose falsification costs the most, under the three cost weightings we tested, while attacks on the inventory sensor change the cost little but raise spoilage, which a cost-only view would miss; how much each attack costs depends on the weights, so this sensitivity analysis should be repeated on a plant’s own model and economic weights to tell operators which sensors to protect first. Second, a closed control loop should be monitored by a detector that predicts the dynamics, not by a detector that judges each reading on its own: the feedback it is meant to protect keeps falsified readings in their normal range, in simulation and on real plant data. The Kalman filter already present in the IoT-MPC architecture provides such a predictor at almost no extra cost, and a predictor learned from normal data can replace it when no model is available. Third, residual detection limits attacks by an adversary unaware of the detector, but not by one who knows it: an attacker who knows the detector can cause large undetected damage through a single sensor. Fourth, when process uncertainty is high, more data do not improve security. Faster or more precise sensing leaves small attacks invisible and can make them more harmful, whereas a redundant sensor on the most valuable measurement, over an independent network path, sharply limits what even a detector-aware attacker can achieve undetected. One extra meter is a modest investment, but its value turns negative if both meters can be falsified through a shared gateway, because the filter then trusts the falsified data more.

**Security versus performance.** The architecture comparison of [1] recommended IoT data with Kalman filtering (Scenario C) because it performs best in normal operation. Our results qualify this recommendation rather than overturn it. Scenario C is also the architecture most exposed to consistent falsified data, such as replay, while the model-only Scenario A cannot be attacked through its sensors at all. With detection and mitigation, Scenario C keeps its advantage in normal operation and limits the damage of detectable attacks, but the damage from undetected attacks remains and depends on the sensing design.

**Network-level and process-level detection.** The detectors of Section 2 that inspect IoT traffic and the process-level detector studied here see different attacks. Network monitoring can flag unknown devices, flooding, jamming, malformed frames, and frames replayed at the protocol level (for example with reused LoRaWAN frame counters), which covers denial of service and injection by an outsider without keys. It cannot see a correctly authenticated packet that carries a false value from a compromised sensor, gateway or edge node, which is where the process-level detector works; conversely, the process-level detector cannot tell a falsified reading from a sensor fault. The two are best combined: a network alarm on a sensor’s path can lower the process threshold for that sensor, a process alarm can trigger network forensics on the path of the flagged sensor, and the independence of the network paths on which the redundancy result depends is a property that network monitoring can verify. Denial of service illustrates the division of labor: lost readings are visible without any detector and the filter tolerates them, so they are a matter for the network layer.

**Choice of detector.** Our results agree with [13,31] that a stateful CUSUM limits stealthy attackers better than a statistic with short memory when the model is accurate, and add that its advantage against fixed-shape attacks disappears once the relative parameter error reaches about 10%, or when the process noise is underestimated, and that thresholds must be calibrated on the plant itself. Checking the whiteness, scale and tails of the normalized residuals on held-out normal data is a simple way to decide; on HAI, residuals that were white but heavy-tailed also called for the bounded-memory statistic.

**Limitations.** The production plant is the linear model of [1] with simulated parameters and an unconstrained, saturated MPC, so the magnitudes reported here are illustrative, and conclusions may differ for a constrained MPC with active constraints. The real-data validation covers the detection method, not the perishable-goods model or the mitigation, and the HAI attacks act partly on setpoints and commands rather than only on sensor data. We consider attacks on sensor data only, not on actuator commands or the controller. The controller’s inputs are at their lower bound most of the time, which makes the cost respond nonlinearly to large attacks; a controller with different targets or constraints may respond differently. The detector-aware attackers are greedy: they maximize each reading’s deviation rather than planning over the horizon, so an optimal attacker could do more, in particular against the redundant design. The ramping targets act as a natural excitation against replay in the sense of [10]; a replay aligned with them evades detection with noisy sensors but does little damage, and a replay designed jointly with the detector-aware strategy could do more. The detectability analysis of Section 5.2 assumes the filter model is exact and uses its steady-state gain; with model error, the weak directions may shift. Finally, the statistical uncertainty of the stealthy damage is large, and some differences between sensing designs are within it.

**Deployment.** Several questions arise only in a real plant. Thresholds must be calibrated on the plant’s own normal data, including its seasonal and production changes, and recalibrated when the process or the sensors change. Over months of continuous operation, permanent isolation would eventually remove sensors after false alarms, so a supervised re-admission procedure and a maintenance check of isolated sensors are needed. The detector also needs a model good enough for its residuals to be nearly white, which takes system identification and validation effort. Finally, an attacker who observes the plant for long enough may learn the model and the detector, so the imperfect-knowledge results of Section 5.6 describe a window of protection rather than a permanent one.

**Future work.** Detecting slow ramps with a single sensor remains the weakest point; tests matched to the drift signature of a ramp, such as generalized likelihood ratio tests on the innovation sequence, may shorten detection delays. Other directions are an optimal, rather than greedy, stealthy attacker and the corresponding bounds [26,33], adaptive attackers that learn the detector online and defenses that change their thresholds or add watermarking in response [10], attacks on actuators, an experimental evaluation of combined network-level and process-level detection, and validation of the plant model and the redundancy recommendation on operational data from a food or pharmaceutical producer.

## 7. Conclusions

IoT-enabled MPC improves the operation of perishable-goods production, but it makes production decisions depend on sensor data that an attacker can falsify. This study evaluated, on the published model of such a plant and on a real industrial testbed, how much damage sensor attacks cause and how far a defense built from the existing Kalman filter limits it. The results support four contributions.

First, the attack surface can be ranked: falsifying one energy meter raised the tracking cost by 71% and replaying old readings raised spoilage by 27%, and a transfer-function analysis of the filter predicted in advance which sensors are hard to protect. Second, the defense (a per-sensor CUSUM test, isolation of flagged sensors and a rewind-and-refilter repair) detected these attacks in 89% and 100% of runs at a nominal 1% false-alarm rate and limited the cost increase to 9% and 3%, at a computational cost of a few thousand operations per reading; its power fell with sensor noise, packet loss and model error, and a bounded-memory statistic degraded more slowly under model error. Third, static detectors missed moderate and slow attacks in closed loop, while predictive detectors found them, in simulation and on the HAI testbed, where a residual detector built from normal data found 32 of 34 attacks at 0.24 false alarms per day, against 27 for a windowed autoencoder and 21 for a static range check. Fourth, detection has limits that the sensing design can shift: a fully informed attacker raised the cost by 93 to 121% undetected, errors of 5 to 20% in the attacker’s model made the same attack detectable in 36 to 74% of runs in Experiment 1, and a redundant meter on an independent path limited the undetected damage to about 15%, whereas a redundant meter reachable through the same gateway increased it.

For practitioners, the recommendation is to rank sensors by the damage their falsification can cause under the plant’s own cost weights, to monitor them with a detector that predicts the dynamics and is calibrated on the plant, to choose CUSUM or a bounded-memory statistic according to how well the model fits, to add a per-reading test against intermittent attacks, and to duplicate the most valuable sensor over an independent path.

## Figures and Tables

**Table 1:** Reproduction of the published baseline: mean total cost ± standard error over 100 Monte Carlo runs, no attack. Published standard errors are computed from the standard deviations reported in [1].

	Exp. 1 (Good Sensors)			Exp. 2 (Noisy, Sparse Sensors)		
	A	B	C	A	B	C
Published [1]	51.64 ± 1.67	59.96 ± 1.16	51.21 ± 1.12	65.95 ± 2.52	105.90 ± 2.89	59.04 ± 1.63
This study	53.26 ± 1.58	58.57 ± 1.13	48.78 ± 0.96	63.97 ± 2.41	106.54 ± 2.98	62.88 ± 1.88

**Table 2:** Levels of attacker knowledge considered in this paper.

Attacker	Plant Dynamics	Detector Parameters	Filter and Detector State	Readings Observed
Fixed-shape	which sensor feeds which state	none	none	clean readings of its sensors
Detector-aware, own channel	exact model	exact τ, ν	exact, own sensor	clean readings of its sensors
Detector-aware, full bank	exact model	exact τ, ν	exact, all sensors	all readings
Imperfect knowledge	model with relative error $\epsilon_{a}$	estimate $\tau_{a}=f\tau$	own replica only	readings and commands in transit

**Table 3:** Mean tracking cost (100 runs) under attack. A: model only; B: raw IoT; C: IoT with Kalman filter; C+D: with detection and mitigation. $P_{D}$ (with 95% Wilson interval) and delay refer to C+D; delays are medians over detected runs with interquartile ranges, measured from attack onset. Spoilage is the mean spoilage rate. The standard error of mean costs is about 1 in Experiment 1 and about 2 in Experiment 2.

	Attack	A	B	C	C+D	$P_{D}$	95% CI	Delay (h)	Spoilage C	Spoilage C+D
Exp. 1	None	53.3	58.6	48.8	48.8	0.01 ^†^	[0.00, 0.05]	–	1.051	1.051
	Energy bias −1.0	53.3	98.7	83.6	53.2	0.89	[0.81, 0.94]	0.3 [0.2, 0.4]	1.090	1.055
	Inventory bias −1.0	53.3	57.8	48.5	48.9	0.29	[0.21, 0.39]	0.3 [0.2, 0.3]	1.119	1.095
	Spoilage bias −1.0	53.3	59.9	48.6	48.9	0.98	[0.93, 0.99]	0.2 [0.1, 0.2]	1.098	1.055
	Energy ramp −0.2/h	53.3	82.5	66.9	65.6	0.15	[0.09, 0.23]	5.2 [4.8, 5.8]	1.066	1.066
	Replay (all sensors)	53.3	99.9	111.0	50.1	1.00	[0.96, 1.00]	0.5 [0.5, 0.6]	1.330	1.075
	DoS (80% loss)	53.3	55.3	49.7	49.7	–	–	–	1.075	1.075
Exp. 2	None	64.0	106.5	62.9	63.0	0.02 ^†^	[0.01, 0.07]	–	1.012	1.013
	Energy bias −1.0	64.0	155.5	83.7	83.0	0.10	[0.06, 0.17]	3.5 [2.3, 5.1]	1.033	1.032
	Inventory bias −1.0	64.0	106.5	62.4	62.4	0.01	[0.00, 0.05]	4.5 (1 run)	1.061	1.061
	Spoilage bias −1.0	64.0	108.2	62.6	62.7	0.35	[0.26, 0.45]	3.3 [2.3, 4.5]	1.027	1.025
	Energy ramp −0.2/h	64.0	133.6	71.4	71.3	0.07	[0.03, 0.14]	5.7 [4.7, 6.1]	1.019	1.019
	Replay (all sensors)	64.0	139.9	83.4	73.8	1.00	[0.96, 1.00]	3.5 [2.7, 4.3]	1.151	1.105
	DoS (80% loss)	64.0	90.5	61.1	61.1	–	–	–	1.017	1.017

^†^ False-alarm rate. DoS is observable directly from missing packets and is not a residual-detection problem.

**Table 4:** Detectability of a constant bias on each sensor from the mean innovation (10), per unit bias and in innovation standard deviations: at the first reading after onset, accumulated over 3 h, and in steady state; the persistent bias $\nu /G_{ii}\left(1\right)$ below which the steady-state innovation stays under the CUSUM drift; the alarm time predicted by the noise-free CUSUM (“Predicted”) for a bias of 1.0 (Exp. 1) or 2.0 (Exp. 2); and the simulated detection probability for the same bias.

	Sensor	Onset	3 h Total	Steady State	$\nu /G_{\mathrm{ii}}$ (1)	Predicted	Simulated $P_{D}$
Exp. 1	Inventory	3.63	2.5	0.011	47	none	0.29
	Energy	3.83	16.9	0.066	7.5	0.5 h	0.89
	Spoilage	5.55	20.2	0.214	2.3	0.3 h	0.98
Exp. 2	Inventory	0.87	1.2	0.030	16.5	none	0.00
	Energy	1.01	4.7	0.095	5.3	none	0.65
	Spoilage	1.39	6.2	0.320	1.6	2.0 h	0.97

**Table 5:** Detector-aware attackers (Section 3.3). Cost increase relative to the attack-free cost without and with the defense, with 95% paired bootstrap interval for the undefended run; $P_{D}$ counts alarms on any sensor; mean spoilage without defense (attack-free: 1.051 in Exp. 1, 1.012 in Exp. 2). CUSUM thresholds recalibrated on 1000 fresh attack-free runs (τ=9.0 and 6.7); per-reading limit $\tau_{1}$ set for 0.5% false alarms with CUSUM retuned so that the pair keeps a 1% rate; windowed $\chi^{2}$ thresholds of Appendix C. The full-bank attacker on the energy meter gives the same results as the own-channel attacker in both experiments.

Setting	Target and Attacker	Detector	Undefended	Defended	$P_{D}$	Spoilage
Exp. 1	Energy meter, own channel	CUSUM	+121% [113, 129]	+121%	0.01	1.098
	Energy meter, own channel	CUSUM + per-reading	+124% [117, 132]	+124%	0.01	1.097
	Energy meter, own channel	Windowed $\chi^{2}$, W=10	+361% [343, 381]	+361%	0.01	1.132
	Energy meter, own channel	Windowed $\chi^{2}$, W=5	+541% [513, 568]	+541%	0.01	1.152
	RFID inventory, own channel	CUSUM	+31% [27, 35]	+31%	0.25	1.287
	RFID inventory, full bank	CUSUM	+30% [27, 34]	+30%	0.25	1.286
Exp. 2	Energy meter, own channel	CUSUM	+93% [86, 101]	+93%	0.01	1.059
	Energy meter, own channel	CUSUM + per-reading	+97% [89, 105]	+98%	0.01	1.060
	Energy meter, own channel	Windowed $\chi^{2}$, W=10	+197% [186, 208]	+196%	0.00	1.079
	Energy meter, own channel	Windowed $\chi^{2}$, W=5	+201% [189, 214]	+201%	0.00	1.078
	RFID inventory, own channel	CUSUM	+31% [26, 36]	+31%	0.10	1.327
	RFID inventory, full bank	CUSUM	+20% [17, 24]	+20%	0.05	1.309

**Table 6:** Run-level recall and F1 score (equal numbers of attacked and attack-free runs, 1% false alarms per run for every detector; 100 runs per cell). Range check: open-loop and closed-loop study of Section 5.3; –: not evaluated. Bold: best detector in each column within each experiment.

		Bias −1.0		Bias −2.0		Ramp −0.3/h		Replay	
	Detector	Recall	F1	Recall	F1	Recall	F1	Recall	F1
Exp. 1	Kalman filter + CUSUM	**0.89**	**0.94**	**1.00**	**1.00**	0.66	0.79	**1.00**	**1.00**
	Kalman filter + windowed $\chi^{2}$	0.68	0.80	0.99	0.99	0.01	0.02	0.99	0.99
	Neural predictor + CUSUM	0.46	0.63	**1.00**	**1.00**	**0.70**	**0.82**	0.99	0.99
	Linear predictor + CUSUM	0.44	0.61	**1.00**	**1.00**	0.23	0.37	**1.00**	**1.00**
	Range check	0.14	0.24	0.91	0.95	0.03	0.06	–	–
	Isolation Forest	0.00	0.00	0.00	0.00	0.00	0.00	0.00	0.00
Exp. 2	Kalman filter + CUSUM	**0.10**	**0.18**	**0.65**	**0.78**	**0.24**	**0.38**	**1.00**	**1.00**
	Kalman filter + windowed $\chi^{2}$	0.00	0.00	0.02	0.04	0.00	0.00	0.64	0.78
	Neural predictor + CUSUM	0.05	0.09	0.46	0.63	0.14	0.24	0.98	0.98
	Linear predictor + CUSUM	0.01	0.02	0.33	0.49	0.06	0.11	**1.00**	**1.00**
	Isolation Forest	0.00	0.00	0.00	0.00	0.00	0.00	0.00	0.00

**Table 7:** Sensing designs under the process noise of Experiment 2 (detector recalibrated to 1% false alarms in each; fixed-shape attacks on the energy meter; 100 runs per design). “Cost, bias −1.0”: undefended cost increase. With redundant meters, an alarm on either meter isolates both (used for all redundant results in Table 7 and Table 8). Bold: the redundant designs and the values in which they are better than all other designs.

Design	$J_{0}$	$P_{D}$ (Bias −1.0)	Cost, Bias −1.0	Stealthy Damage	Worst-Case Defended	$P_{D}$ (Ramp −0.3/h)
Baseline: 3 sensors, every 4th step	62.9	0.09	+33%	22%	50%	0.22
+ machine-vibration sensor	61.8	0.13	+32%	23%	59%	0.24
Sample every 2nd step	61.1	0.10	+40%	28%	55%	0.27
Sample every step	56.6	0.10	+50%	36%	57%	0.14
+ machine sensor, every step	60.7	0.18	+44%	19%	46%	0.18
Sensors 4× more precise	61.2	0.15	+47%	18%	58%	0.38
Sensors 16× more precise	60.7	0.20	+55%	21%	57%	0.46
**Redundant energy meter**	60.4	0.31	**+17%**	**8%**	**19%**	0.58
**Redundant energy meter, every step**	58.0	**0.86**	**+22%**	**5%**	**15%**	**0.99**

**Table 8:** Energy-meter redundancy against stronger attackers (process noise of Experiment 2, 100 runs, thresholds of Table 7). Cost increase relative to the attack-free cost of each design, without defense and with the defense (isolating both meters on an alarm); $P_{D}$ of the undefended run, alarms on any sensor. Full bank ($\bar{z}=0.4$): the attacker also keeps the second meter’s predicted normalized innovation below 0.4.

Design	Attack	$P_{D}$	Undefended	Defended
Single meter, every 4th step	Bias −1.0	0.09	+33%	+32%
	Detector-aware, own channel	0.01	+93%	+93%
Redundant meter, every 4th step	Bias −1.0, one meter	0.31	+17%	+16%
	Detector-aware, own channel, one meter	0.21	+25%	+26%
	Detector-aware, full bank, one meter	0.20	+25%	+26%
	Detector-aware, full bank ($\bar{z}=0.4$), one meter	0.03	+15%	+15%
	Bias −1.0, both meters	0.12	+42%	+40%
	Bias −0.5, both meters	0.01	+17%	+17%
	Detector-aware, full bank, both meters	0.01	+167%	+167%
Single meter, every step	Detector-aware, own channel	0.00	+169%	+169%
Redundant meter, every step	Bias −1.0, one meter	0.86	+22%	+14%
	Detector-aware, own channel, one meter	0.48	+20%	+20%
	Detector-aware, full bank, one meter	0.45	+20%	+20%
	Detector-aware, full bank ($\bar{z}=0.4$), one meter	0.00	+10%	+10%
	Bias −1.0, both meters	0.08	+57%	+52%
	Bias −0.5, both meters	0.00	+22%	+22%
	Detector-aware, full bank, both meters	0.00	+297%	+297%

**Table 9:** Requirements and benefits of the sensing designs (process noise of Experiment 2). Readings per hour count all sensors. Stealthy damage: fixed-shape attacks detected in at most 5% of runs; worst-case defended: largest damage with the defense active; detector-aware: own-channel attacker on one energy meter, undetected. –: not evaluated.

Design	Added Hardware	Readings/h	$P_{D}$ (Bias −1.0)	Stealthy Damage	Worst-Case Defended	Detector-Aware
Baseline: 3 sensors, every 4th step	none	7.5	0.09	22%	50%	93%
+ machine-vibration sensor	1 sensor	10	0.13	23%	59%	–
Sample every step	none	30	0.10	36%	57%	169%
Sensors 16× more precise	3 better sensors	7.5	0.20	21%	57%	–
Redundant energy meter	1 m	10	0.31	8%	19%	15–25%
Redundant energy meter, every step	1 m	40	0.86	5%	15%	10–20%

**Table 10:** Detection and defended damage under model mismatch (energy bias −1.0 and replay). ϵ: relative error of the plant parameters (mean over 20 plants, each calibrated on 300 of its own attack-free runs and evaluated on 50 more); exact model: 5 calibrations of 300 runs, each evaluated on 50 runs; noise rows: calibration on 500 runs, evaluation on 100; $Q_{w}\times 2$ etc.: true noise variance relative to the filter’s. FA: false-alarm rate of the thresholds calibrated on the nominal model; $P_{D}$ and defended cost increase after recalibration on the mismatched plant; C: CUSUM, $\chi^{2}$: windowed statistic (W=10). Undefended cost increase under replay: +120 to +137% (Exp. 1) and +31 to +37% (Exp. 2). Bold: the better of the two detectors in each case.

		FA (Nominal)		$P_{D}$, Bias		Bias,	Bias, Defended		$P_{D}$, Replay		Replay, Defended	
	Case	C	$\chi^{2}$	C	$\chi^{2}$	Undefended	C	$\chi^{2}$	C	$\chi^{2}$	C	$\chi^{2}$
Exp. 1	Exact model	0.01	0.01	**0.84**	0.68	+72%	**+11%**	+23%	1.00	1.00	**+4%**	+6%
	ϵ=0.1	0.06	0.01	0.58	**0.66**	+71%	+32%	**+25%**	0.99	1.00	+12%	+11%
	ϵ=0.2	0.41	0.07	0.24	**0.51**	+66%	+53%	**+34%**	0.99	0.99	+46%	**+26%**
	$Q_{w}\times 2$	0.35	0.05	0.13	**0.51**	+63%	+55%	**+31%**	1.00	0.99	+14%	+13%
	$Q_{w}\times \frac{1}{2}$	0.00	0.01	**1.00**	0.67	+78%	**+3%**	+27%	0.99	0.99	+2%	+1%
	$R_{v}\times 2$	0.10	0.78	**0.64**	0.08	+72%	**+26%**	+67%	1.00	0.99	**+8%**	+37%
	$R_{v}\times \frac{1}{2}$	0.01	0.00	0.85	**0.99**	+72%	+12%	**+4%**	1.00	0.99	+1%	+3%
Exp. 2	Exact model	0.01	0.01	0.13	0.00	+35%	+34%	+35%	**1.00**	0.67	**+18%**	+33%
	ϵ=0.1	0.07	0.01	0.06	0.00	+33%	+33%	+33%	**0.98**	0.66	**+24%**	+31%
	ϵ=0.2	0.34	0.05	0.03	0.00	+31%	+31%	+31%	0.72	0.59	+31%	+32%
	$Q_{w}\times 2$	0.16	0.02	0.08	0.00	+26%	+26%	+26%	**0.98**	0.39	+27%	+31%
	$Q_{w}\times \frac{1}{2}$	0.00	0.01	0.11	0.00	+37%	+34%	+36%	**1.00**	0.74	**+13%**	+37%
	$R_{v}\times 2$	0.12	0.37	0.04	0.00	+32%	+31%	+32%	**1.00**	0.02	**+25%**	+33%
	$R_{v}\times \frac{1}{2}$	0.00	0.00	0.14	0.01	+34%	+33%	+33%	**1.00**	0.98	**+13%**	+24%

**Table 11:** Intermittent energy-meter bias of −1.0 in Experiment 1 (period *T*, duty cycle *d*; 100 runs). Detection probability and cost increase with the defense; undefended cost increases in the last column.

		CUSUM		Windowed $\chi^{2}$		CUSUM + Per-Reading		
T (h)	d	$P_{D}$	Defended	$P_{D}$	Defended	$P_{D}$	Defended	Undefended
–	1 (persistent)	0.89	+9%	0.68	+22%	0.81	+14%	+71%
1	0.5	0.99	+6%	0.99	+3%	1.00	+7%	+41%
1	0.25	0.86	+13%	0.99	+4%	0.97	+8%	+31%
1	0.1	0.02	+12%	0.69	+9%	0.93	+5%	+12%
2	0.25	1.00	+5%	0.99	+3%	1.00	+6%	+33%
0.4	0.5	0.37	+20%	0.99	+2%	0.92	+10%	+28%

**Table 12:** Detector-aware attacker on the energy meter with imperfect knowledge (100 runs; CUSUM thresholds 8.5 and 6.5). $\epsilon_{a}$: relative error of the attacker’s model; *f*: assumed threshold relative to the true one. Cells: $P_{D}$ / undefended / defended cost increase. Fully informed attacker: $P_{D}=0.01$, +116% (Exp. 1) and +89% (Exp. 2).

	$\epsilon_{a}$	f=0.8	f=1.0	f=1.2
Exp. 1	0	0.06 / +111% / +104%	0.26 / +118% / +88%	1.00 / +132% / +2%
	0.05	0.36 / +114% / +85%	0.67 / +124% / +49%	1.00 / +135% / +2%
	0.1	0.47 / +119% / +66%	0.72 / +129% / +36%	1.00 / +143% / +2%
	0.2	0.57 / +143% / +49%	0.74 / +157% / +27%	1.00 / +171% / +2%
Exp. 2	0	0.06 / +79% / +76%	0.29 / +94% / +67%	1.00 / +113% / +4%
	0.05	0.21 / +80% / +73%	0.53 / +98% / +58%	1.00 / +116% / +4%
	0.1	0.30 / +82% / +68%	0.57 / +97% / +50%	1.00 / +117% / +4%
	0.2	0.44 / +89% / +56%	0.59 / +104% / +40%	1.00 / +125% / +4%

**Table 13:** Detection of the 34 boiler attacks in HAI 21.03, with thresholds fixed on validation data for one false-alarm episode per day. False alarms are counted on the attack-free parts of the test data. FA/day: false-alarm episodes per day; alarm time: share of attack-free time with the alarm on; PV masked: attacks in which the process variable (sensor value) was masked; delay: median detection delay; onset rule: the alarm must switch on between the start of the attack and 60 s after its end. eTaP and eTaR are the precision and recall parts of the eTaPR score; its F1 scores [22] use $\theta_{p}=0.5$, δ=0 and $\theta_{r}=0.01$, 0.1 or 0.5.

Detector	Detected	Onset Rule	PV Masked	Not Masked	FA/Day	Alarm Time	Delay	eTaP/eTaR/ F1 ($\theta_{r}=0.01$)	F1 ($\theta_{r}=0.5$)
ARX residual, windowed $\chi^{2}$	**32/34**	27/34	15/16	17/18	**0.24**	**0.01%**	6 s	0.69/**0.52**/**0.59**	**0.57**
Neural predictor residual, windowed $\chi^{2}$	28/34	26/34	14/16	14/18	0.48	0.03%	20 s	0.67/0.45/0.54	0.52
Windowed autoencoder (raw signals)	27/34	27/34	15/16	12/18	0.24	<0.001%	32 s	**0.78**/0.40/0.53	0.42
ARX residual, CUSUM (ν=8) ^†^	33/34	28/34	16/16	17/18	1.7	0.85%	4 s	0.20/0.24/0.22	0.17
ARX residual, CUSUM (ν=0.5)	30/34	13/34	15/16	15/18	11.0	51%	0 s	0.00/0.00/0.00	0.00
Static range check (raw signals)	21/34	21/34	13/16	8/18	0.24	0.007%	51 s	0.74/0.26/0.39	0.36
Isolation Forest (raw signals)	2/34	2/34	2/16	0/18	0.24	0.005%	–	0.63/0.02/0.05	0.03

^†^ Best drift of a grid evaluated on the test data. A CUSUM alarm is often already on when an attack starts, hence the gap to the onset rule.

## Data Availability

The simulation code, the Python 3.13 port of the published model, and the result files supporting the findings of this study are available from the author upon reasonable request. The HAI 21.03 dataset analyzed in Section 5.7 is publicly available at https://github.com/icsdataset/hai (accessed on 20 October 2026) under the CC BY-SA 4.0 license.

## Abbreviations

The following abbreviations are used in this manuscript:

ARX	Autoregressive with exogenous input
CI	Confidence interval
CUSUM	Cumulative sum
DCS	Distributed control system
DoS	Denial of service
eTaP, eTaR	Precision and recall parts of eTaPR
eTaPR	Enhanced time-series aware precision and recall
EWMA	Exponentially weighted moving average
FAR	False-alarm rate
HAI	Hardware-in-the-loop-based augmented industrial control system (dataset)
IoT	Internet of Things
LoRaWAN	Long-range wide-area network
MPC	Model predictive control
OPC UA	OPC Unified Architecture
PTI	Predictive technology intervention
PV	Process variable (sensor value)
QP	Quadratic program
ReLU	Rectified linear unit
RFID	Radio-frequency identification
SWaT	Secure Water Treatment (dataset)
USAD	Unsupervised anomaly detection (autoencoder method)

## Appendix A. Model and Controller Parameters

The parameters below (Table A1) are those of the published model and code [1]. Continuous-time dynamics (states in the normalized units of [1]):

$$\begin{array}{cccccc} \dot{I} & =-\theta I-F+P-S, & \dot{M} & =-\alpha_{1}M+\alpha_{2}E+\alpha_{3}u_{1}, & \dot{E} & =\beta_{1}I+\beta_{2}M-\beta_{3}E+\beta_{4}u_{2},\\ \dot{F} & =-\delta F+u_{3}, & \dot{P} & =\gamma_{2}F-\gamma_{1}P+u_{4}, & \dot{A} & =-\kappa A+u_{5},\\ \dot{S} & =\phi_{2}I+\phi_{3}E-\phi_{4}A-\phi_{1}S. & \; & & \; \end{array}$$

**Table A1:** Model parameters, weights and targets.

Quantity	Value
Dynamics	θ=0.05, $\alpha_{1}=0.04$, $\alpha_{2}=0.02$, $\alpha_{3}=0.3$, $\beta_{1}=0.01$, $\beta_{2}=0.05$, $\beta_{3}=0.1$, $\beta_{4}=0.8$,
	δ=0.2, $\gamma_{1}=0.1$, $\gamma_{2}=0.1$, κ=0.5, $\phi_{1}=0.15$, $\phi_{2}=0.04$, $\phi_{3}=0.03$, $\phi_{4}=0.1$
Initial state	$x_{0}={\left[5,2,4,3,1,2,1\right]}^{⊤}$; filter initialized at $x_{0}$ with $\Sigma_{0}=0.1\;I$
Stage weights	Q=diag(0.1,2,5,0.4,0.2,0.2,5), $R=0.1\;I_{5}$
Terminal weights	$P_{T}=\mathrm{diag}\left(50,\;50,\;150,\;50,\;50,\;50,\;150\right)$
MPC	horizon m=5; block weights 2Q for steps 1 to m−1 and $\left(2/h\right)P_{T}+Q$ for step *m*;
	*R* for the first input and 2R for the others; Hessian $H=hR_{\mathrm{blk}}+h^{3}\Upsilon^{⊤}Q_{\mathrm{blk}}\Upsilon +10^{-2}I$
Gain	$K_{\mathrm{MPC}}$ is the first block row of $-H^{-1}h\;\Upsilon^{⊤}Q_{\mathrm{blk}}\Gamma$, with $\Gamma =[A_{d};A_{d}^{2};\ldots ;A_{d}^{m}]$
	and ${\left[\Upsilon \right]}_{ij}=A_{d}^{\;i-j}B_{d}$ for j≤i (zero otherwise); $Q_{\mathrm{blk}},R_{\mathrm{blk}}$: block weights above
Input bounds	$0\leq u_{j}\leq 10$
Targets	$I^{\mathrm{ref}}=5+0.5t$, $M^{\mathrm{ref}}=2$, $E^{\mathrm{ref}}=4+\sin\left(0.5\pi t\right)$, $F^{\mathrm{ref}}=3e^{-0.1t}$,
	$P^{\mathrm{ref}}=1+0.2t$, $A^{\mathrm{ref}}=2\cdot ⊮\left(t<5\right)$, $S^{\mathrm{ref}}=1+0.1t$ (*t* in hours)
Sampling	h=0.1 h, horizon 10 h (101 steps)

The factors *h*, $h^{3}$ and $\left(2/h\right)P_{T}$ come from the discretized quadratic cost of the original code of [1], which this port reproduces.

## Appendix B. Main Symbols

Table A2 lists the main symbols used in the paper.

**Table A2:** Main symbols. Plant states use the names of [1]; matrices are distinguished from states by subscripts or by context.

Symbol	Meaning
I,M,E,F,P,A,S	states: inventory, machine performance, energy, forecast, production, PTI level, spoilage
$x_{k}$, $u_{k}$, $y_{k}$	state, input and reading vectors at step *k*
$A_{d}$, $B_{d}$, *C*	discretized plant matrices and sensor selection matrix
*h*	simulation step (0.1 h); $n_{s}$: steps between readings
$Q_{w}$, $R_{v}$	process and measurement noise covariances
*Q*, *R*, $P_{T}$	stage and terminal weights of the MPC; *J*: tracking cost (5)
$\Sigma_{k|k-1}$, $V_{k}$, $K_{k}$	predicted error covariance, innovation covariance, Kalman gain
$r_{k}$, $z_{i,k}$	innovation vector and normalized innovation of sensor *i*
$g_{i,k}^{\pm }$, ν, τ	CUSUM statistics, drift and threshold
$s_{i,k}$, *W*, $\tau_{\chi }$	windowed $\chi^{2}$ statistic, window length and threshold; $\tau_{1}$: per-reading threshold
$t_{a}$, $k_{a}$, ${\hat{k}}_{a}$	attack start time and step, estimated onset
*b*, *s*	bias magnitude and ramp slope
$P_{D}$, FAR	detection probability and false-alarm rate per run
$D_{\varepsilon }$	stealthy damage (6)
ϵ, $\epsilon_{a}$, *f*	plant-parameter error; attacker’s model error; attacker’s assumed threshold factor
G(q)	transfer function from a sensor attack to the mean innovation (10)

## Appendix C. Detector Settings and Alarm Attribution

Table A3 lists the settings of all detectors and baselines. Table A4 separates, for the fixed-shape attacks of Table 3, alarms on the attacked sensor from alarms on other sensors, removes runs whose attack-free twin (same seed) also raises an alarm after the attack time, and reports how often the defense isolated only uncompromised sensors.

**Table A3:** Detector and baseline settings. All thresholds give 1% false alarms per 10-h run in simulation (on attack-free runs with seeds separate from the evaluation seeds) or one false-alarm episode per day on the HAI validation data.

Detector	Settings
Kalman filter + CUSUM	ν=0.5; τ=8.5 (Exp. 1) and 6.5 (Exp. 2) from 1000 runs; recalibrated per sensing design (e.g., 7.24 and 9.68 for the redundant meter every 4th step and every step)
CUSUM + per-reading test	$\tau_{1}=17.9$ and 15.6 (0.5% false alarms alone), with τ=9.5 and 7.0 (Exp. 1, Exp. 2)
Windowed $\chi^{2}$ (simulation)	at least 3 readings; $\tau_{\chi }=3.93$ and 4.07 for W=10, 5.29 and 4.78 for W=5, 3.75 and 4.07 for W=20 (Exp. 1, Exp. 2)
Range check	per-step mean and standard deviation of each reading from 300 runs; alarm when any |z| exceeds 3.62 (Exp. 1) or 3.03 (Exp. 2) at two consecutive readings
Isolation Forest (simulation)	300 trees, 256 samples per tree, all features; trained on 150 runs; features in normalized plant units; alarm at two consecutive readings above a threshold calibrated on 300 runs; lagged variant uses the last five readings’ deviations and inputs
Learned predictors (simulation)	inputs: last 5 readings and inputs and the current targets, standardized; ridge penalty $10^{-2}$; neural network with two hidden layers of 64 ReLU units, Adam, early stopping on 10% of the data, at most 400 epochs; trained on 300 runs, residual scales from 500 runs; CUSUM with ν=0.5
HAI: data split	training: train1, train2 and the first 70% of train3; held-out validation (normalization of residuals and thresholds): last 30% of train3
HAI: signals	inputs: 8 setpoints (P1_B2004, B2016, B3004, B3005, B4002, B4005, B400B, B4022), 5 valve commands (P1_FCV01D, FCV02D, FCV03D, LCV01D, PCV01D) and 17 sensors (P1_FCV01Z, FCV02Z, FCV03Z, LCV01Z, PCV01Z, PCV02Z, FT01, FT01Z, FT02, FT02Z, FT03, FT03Z, LIT01, PIT01, PIT02, TIT01, TIT02), standardized with training statistics; targets: the 22 commands and sensors
HAI: ARX predictor	5 lags, one-second-ahead, ridge penalty $10^{-3}$; windowed $\chi^{2}$ over 60 s, threshold 201; CUSUM thresholds 423 (ν=0.5) and 89.9 (ν=8)
HAI: neural predictor	hidden layers of 128 and 64 ReLU units, Adam (learning rate $10^{-3}$, batch 512), early stopping, at most 60 epochs, 120,000 random samples per training file; windowed $\chi^{2}$ threshold 284
HAI: Isolation Forest	200 trees, 200,000 random training samples of the standardized signals and their 60-s means; score averaged over 60 s; threshold 0.612

For the detector-aware attacks of Table 5 and Table 8, no alarm was ever raised on an attacked sensor: every alarm came from another sensor whose innovations responded to the corrupted estimate. With a single meter, these alarms isolate the wrong sensor (in 25% of runs for the inventory attack in Experiment 1); with redundant meters, isolating both meters on an alarm turns them into a correct response.

**Table A4:** Alarm attribution for the fixed-shape attacks of Table 3 (100 runs). $P_{D}$ counts the first alarm on any sensor; “attacked sensor” requires it on an attacked sensor; “net” is $P_{D}$ (any sensor) excluding runs whose attack-free twin also alarms after the attack time (1% of runs in Exp. 1, 2% in Exp. 2); “wrong sensor” is the share of defended runs in which only uncompromised sensors were isolated.

	Attack	$P_{D}$	Attacked Sensor	Net	Wrong Sensor
Exp. 1	Energy bias −1.0	0.89	0.88	0.88	0.01
	Inventory bias −1.0	0.29	0.28	0.28	0.01
	Spoilage bias −1.0	0.98	0.98	0.97	0.00
	Energy ramp −0.2/h	0.15	0.14	0.14	0.01
	Replay	1.00	1.00	0.99	0.00
Exp. 2	Energy bias −1.0	0.10	0.09	0.09	0.01
	Inventory bias −1.0	0.01	0.01	0.00	0.00
	Spoilage bias −1.0	0.35	0.32	0.33	0.02
	Energy ramp −0.2/h	0.07	0.06	0.05	0.01
	Replay	1.00	1.00	0.98	0.00

## Appendix D. Additional Simulation Results

**Accuracy of the onset estimate.** The rewind step needs the attack onset ${\hat{k}}_{a}$ (Section 4.4). For biases and replay the estimate is early, by a median of 0.8 h (interquartile range 0.3 to 1.6 h) for the energy bias in Experiment 1 and 0.65 h for replay, because the CUSUM statistic is seldom exactly zero just before the attack. For the slow ramp it is late, by a median of 1.2 h, because the statistic starts to grow only once the offset is large. An early estimate discards some valid readings and a late one keeps some falsified ones, but neither matters much: rewinding to the true onset instead lowers the mitigated cost increase from 9% to 7% for the energy bias and from 2.7% to 2.3% for replay, and changes it by less than half a percentage point for the ramp and in Experiment 2.

**Attack timing.** All percentages above depend on when the attack starts, because the cost accumulates over the whole horizon. Table A5 therefore reports the cost increase after the attack starts, for start times from 1 to 7 h. The undefended damage and the detection results hardly depend on the start time in Experiment 1 (82 to 99% after onset, detection in 81 to 90% of runs, median delay 0.3 h). The defended damage varies more (10 to 41%), because what the plant loses with the meter depends on what it is doing when the meter is removed. In Experiment 2, detection falls from 72% to 54% as the attack starts later, since fewer readings remain in which to detect it.

**Table A5:** Energy-meter bias started at different times (−1.0 in Experiment 1, −2.0 in Experiment 2; 100 runs). Cost increase accumulated from the start of the attack to the end of the horizon, relative to the same period without attack; $P_{D}$ and median delay of the undefended run.

	Start (h)	Undefended	Defended	$P_{D}$	Delay (h)
Exp. 1	1	+96%	+28%	0.90	0.3
	2	+82%	+16%	0.81	0.3
	3	+99%	+13%	0.89	0.3
	5	+93%	+41%	0.89	0.3
	7	+90%	+10%	0.90	0.3
Exp. 2	1	+122%	+53%	0.72	2.5
	2	+110%	+52%	0.71	2.3
	3	+118%	+66%	0.65	1.7
	5	+85%	+55%	0.59	2.1
	7	+71%	+49%	0.54	1.7

**Replay variants.** Aligning the replay with the targets removes most of its damage. In Experiment 1 it raises the cost by 9% instead of 128% and is still detected in every run (median delay 0.1 h), because the recorded noise and dynamics do not match the current inputs. In Experiment 2 it is almost never detected (1% of runs) but raises the cost by only 9%, and spoilage from 1.012 to 1.038. Starting the replay at 6 h, so that the recording excludes the start-up period, lowers its damage in Experiment 1 to 47% without affecting detection; in Experiment 2 detection drops from 100% to 78%. Most of the damage of replay therefore comes from the mismatch between recorded and current targets, which is also what makes it detectable: an attacker who removes the mismatch gains stealth but loses most of the damage.

**Window length of the $\chi^{2}$ statistic.** The window length matters for the windowed statistic: with windows of 5, 10 and 20 readings, each calibrated on 1000 runs, it detected the Experiment 1 energy bias in 84%, 68% and 9% of runs (CUSUM: 89%), but the 5-reading window is the most vulnerable to the detector-aware attacker (Table 5).

## Appendix E. Boiler Attacks in HAI 21.03

Table A6 lists the 34 boiler attacks of the HAI 21.03 test data used in Section 5.7, with the results of the ARX residual detector with the windowed $\chi^{2}$ statistic. Targets follow the HAI naming: PC, LC and FC are the pressure, level and flow control loops; SP is a setpoint and CO a controller output (valve command); PV indicates that the process variable (sensor value) was masked; ST marks short, repeated attacks.

**Table A6:** Boiler attacks in the HAI 21.03 test data (start time in 2020, month/day hour:minute; duration in seconds) and detection by the ARX residual detector (delay in seconds; * detected only because the alarm was already on).

Start	Target	Duration	Masked	Detected	Delay
07/07 15:35	PC-SP1	192	no	yes	83
07/07 17:28	FC-SP1	98	no	yes	6
07/07 18:59	LC-CO1	190	no	yes	0
07/09 17:38	PC-SP1PV1	422	yes	yes	68
07/09 18:59	LC-CO1-ST	17	no	yes	1
07/09 20:10	FC-SP1PV1	259	yes	yes	18
07/09 21:15	PC-SST	123	no	yes	3
07/09 23:02	FC-CO1PV1	256	yes	yes	14
07/10 01:33	LC-SP1PV1	261	yes	yes	18
07/10 03:03	LC-SP1	199	no	yes	3
07/10 05:29	PC-CO1PV1	421	yes	no	–
07/10 10:49	LC-CO1PV1	254	yes	yes	10
07/10 12:51	PC-CO1	152	no	yes	9
07/10 15:40	FC-CO1-ST	65	no	yes	0
07/10 18:21	FC-CO1	99	no	yes	1
07/13 13:51	LC-CO1-ST + FC-SP1	132	no	yes *	0
07/13 15:21	PC-SP1PV1 + FC-SP1	421	yes	yes	8
07/13 18:11	PC-CO1 + LC-CO1	189	no	yes *	0
07/13 23:55	PC-SP1	238	no	no	–
07/14 01:51	FC-CO1	110	no	yes *	0
07/14 03:53	LC-CO1PV1	255	yes	yes	0
07/28 12:43	PC-CO1 + LC-SP1PV1	254	yes	yes	6
07/28 13:45	FC-SP1PV1	262	yes	yes	23
07/28 15:57	LC-SP1PV1	263	yes	yes	8
07/28 17:45	LC-CO1PV1	258	yes	yes	6
07/30 11:16	PC-CO1	172	no	yes	23
07/30 13:30	FC-CO1PV1	258	yes	yes	7
07/30 16:05	LC-SP1PV1	256	yes	yes	4
07/30 17:45	FC-CO1	120	no	yes	7
07/30 18:38	LC-SP1	203	no	yes	4
07/31 01:15	FC-CO1-ST + LC-CO1-ST	51	no	yes *	0
07/31 02:01	PC-SP1 + PC-CO1	241	no	yes	5
07/31 09:54	LC-SP1 + LC-CO1PV1	262	yes	yes *	0
07/31 11:20	FC-SP1 + FC-CO1PV1	262	yes	yes	8
